# Providing culturally appropriate mental health first aid to an Aboriginal or Torres Strait Islander adolescent: development of expert consensus guidelines

**DOI:** 10.1186/1752-4458-8-6

**Published:** 2014-01-28

**Authors:** Kathryn J Chalmers, Kathy S Bond, Anthony F Jorm, Claire M Kelly, Betty A Kitchener, AJ Williams-Tchen

**Affiliations:** 1 Mental Health First Aid Australia, Parkville, Victoria, Australia; 2 Melbourne School of Population and Global Health, University of Melbourne, Parkville, Victoria, Australia; 3 School of Psychology, Deakin University, Victoria, Australia

**Keywords:** Aboriginal, Torres Strait Islander, Adolescent, Youth, Mental illness, Mental health, Communication, Delphi method, Expert consensus, Community guidelines

## Abstract

**Background:**

It is estimated that the prevalence of mental illness is higher in Aboriginal and Torres Strait Islander adolescents compared to non-Aboriginal adolescents. Despite this, only a small proportion of Aboriginal youth have contact with mental health services, possibly due to factors such as remoteness, language barriers, affordability and cultural sensitivity issues. This research aimed to develop culturally appropriate guidelines for anyone who is providing first aid to an Australian Aboriginal or Torres Strait Islander adolescent who is experiencing a mental health crisis or developing a mental illness.

**Methods:**

A panel of Australian Aboriginal people who are experts in Aboriginal youth mental health, participated in a Delphi study investigating how members of the public can be culturally appropriate when helping an Aboriginal or Torres Strait Islander adolescent with mental health problems. The panel varied in size across the three sequential rounds, from 37–41 participants. Panellists were presented with statements about cultural considerations and communication strategies via online questionnaires and were encouraged to suggest additional content. All statements endorsed as either *Essential* or *Important* by ≥ 90% of panel members were written into a guideline document. To assess the panel members’ satisfaction with the research method, participants were invited to provide their feedback after the final survey.

**Results:**

From a total of 304 statements shown to the panel of experts, 194 statements were endorsed. The methodology was found to be useful and appropriate by the panellists.

**Conclusion:**

Aboriginal and Torres Strait Islander Youth mental health experts were able to reach consensus about what the appropriate communication strategies for providing mental health first aid to an Aboriginal and Torres Strait Islander adolescent. These outcomes will help ensure that the community provides the best possible support to Aboriginal adolescents who are developing mental illnesses or are in a mental health crisis.

## Background

Australia’s most recent National Mental Health and Wellbeing Survey, carried out in 2007, found that mental illnesses were common, associated with substantial disability, but often not treated [1]. Many people with mental illnesses do not seek help for their mental health problems [1]. Despite improvements in mental health literacy in the Australian public over the past decade or so, there is still potential for mental health literacy gains in the areas of recognition and treatment beliefs for mental illnesses [2].

In 2001 the Mental Health First Aid (MHFA) program was developed in response to the often poor mental health literacy of members of the public, who may lack knowledge about mental illnesses and how they can best be treated [2,3]. MHFA is an educational course designed to teach members of the public the skills to recognise and respond to mental illnesses in another person [4]. MHFA is modelled on physical first aid, and is defined as the early help provided to a person developing a mental health problem or in a mental health crisis. First aid is given until appropriate professional treatment is received or the crisis resolves [4]. The MHFA program teaches first aid for a variety of mental health problems, including depression, anxiety, trauma, psychosis, eating disorders and suicidal behaviour. The MHFA program also teaches participants how to assist a person experiencing problem drug use.

The standard MHFA course has been evaluated with 5 controlled trials, 9 uncontrolled trials and 3 qualitative studies [5]. These evaluations have shown that participants have increased mental health literacy, their attitudes to appropriate treatments become more positive, stigma is reduced, they become more confident in providing support, and they report more supportive behaviour towards others. The MHFA program has separate versions for adults and adults assisting youth, and the adult version has been adapted for Aboriginal^a^ Australians and some non-English speaking immigrant groups. More than 200,000 people have completed a MHFA course in Australia and the course has spread to over 20 other countries.

To increase the evidence base of MHFA, guidelines for mental health first aid strategies have been developed using expert consensus (the Delphi method) [6]. Guidelines for providing mental health first aid have been developed for a number of mental health problems and crises including suicidal ideation [7], psychosis [8], depression [9], problem drinking [10], eating disorders [11], traumatic events [12], and drug use [13]. Guidelines for providing mental health first aid specifically to Aboriginal Australians [14] and to youth [6] have also been developed. The current study sought to develop guidelines for being culturally appropriate when providing mental health first aid to Aboriginal adolescents.

### The role of culture in mental health

The way an individual understands and conceptualises mental health is culturally bound, and yet this is not always considered in mainstream mental health service delivery [15,16]. In Australia, it has been identified that there are more barriers for Aboriginal people than non-Aboriginal people at the stage of seeking help from a mental health service [17]. These include a history of racism and discrimination and resultant lack of trust in mainstream services, misunderstandings caused by cultural and language differences, and inadequate measures to reduce the stigma associated with mental illness [17]. Cultural safety should be a component of any mental health programs with Aboriginal and Torres Strait Islander communities [18]. Recently many countries have recognised the important role that culture plays in the identification, treatment and prevention of mental illness and have specialised cultural adaptations of health education programs [19-22].

### Aboriginal and Torres Strait Islander Australians

In Australia, the diverse groups of Aboriginal and Torres Strait Islander people, who constitute 2.5% of the total Australian population [23], have a higher burden of disease and injury than the general Australian population [24]. A recent review of community studies reported that across seven surveys, Aboriginal adults were consistently found to have a higher prevalence of self-reported psychological distress than the general community [25]. There is limited availability of national data on Aboriginal mental health, partially due to difficulties in data collection. However, the 2008 National Aboriginal and Torres Strait Islander Social Survey (NATSISS) found that 31% of Aboriginal adults (aged 15 years and older) reported high or very high levels of psychological distress in the four weeks prior to interview [26]. This was more than twice the rate for non-Aboriginal Australians [27]. Furthermore, data on suicide deaths from 2001–2010 indicate that the overall rates of suicide for Aboriginal people were found to be twice those of non-Aboriginal Australians [28].

The need to provide culturally appropriate training and education for improving Aboriginal Australians mental health [29], coupled with the success of the MHFA course in increasing help-seeking behaviours, led to the development of a cultural adaptation of the MHFA training program, specifically for assisting Aboriginal and Torres Strait Islander Australians. The Aboriginal and Torres Strait Islander Mental Health First Aid course differs from the general MHFA course in recognising the historical, cultural and political forces that have affected Aboriginal mental health [30]. The program acknowledges that Aboriginal people understand mental health within a cultural framework that is not always compatible with western medicine [29,31]. Additionally, the program acknowledges the complexities that can occur in the use of mental health services by Aboriginal people due to barriers to accessing services that provide culturally sensitive treatment approaches, or fear of accessing mental health services because of historical failings to acknowledge and respect an individual’s cultural beliefs about mental illness [31]. The Aboriginal and Torres Strait Islander Mental Health First Aid program has been evaluated as culturally appropriate and acceptable to Aboriginal people [30]. Additionally, best practice guidelines for providing mental health first aid to Australian Aboriginal people were developed using Delphi consensus studies to further enhance the Aboriginal and Torres Strait Islander Mental Health First Aid course [14]. These studies have been independently evaluated as methodologically rigorous [32]. The best practice guidelines were agreed upon, considered useful and culturally appropriate by Aboriginal mental health experts [14].

### The importance of early intervention

Increasingly, research findings suggest that early intervention can provide long-term benefits by preventing the worsening of mental health problems [33]. Early intervention is most appropriate for helping youth because adolescence is the peak age of onset for a first episode of mental illness, with half of all people who will develop any mental illness having experienced an episode by 18 years of age [34].

The Youth Mental Health First Aid (YMHFA) course is a variation of the standard MHFA course and is designed to improve the mental health literacy of adults who assist adolescents. Initial evaluation suggests that the YMHFA course improves participants’ knowledge, attitudes and helping behaviour [6]. The course content and manual are modified to provide information which is specific to helping adolescents [35]. In addition to the standard course content, YMHFA provides information on eating disorders and how to assist a young person who has been engaging in non-suicidal self-injury. The program emphasises the importance of early intervention to minimise the impact of mental health problems on adolescent development. The current research aims to produce culturally appropriate guidelines that may aid early intervention amongst Aboriginal youth with mental health problems.

### The need for mental health first aid guidelines for helping Aboriginal adolescents

There is a need for culturally appropriate mental health services across the life span of Aboriginal people [25]. In addition to providing MHFA guidelines for helping Aboriginal adults, it is imperative that similar resources for helping young Aboriginal Australians are available [14]. This need is especially apparent given that youth make up a large proportion of the Aboriginal population. In 2006, one-third (38%) of Aboriginal and Torres Strait Islander people were under 15 years of age, compared with 19% of non-Aboriginal people [36].

Though there is limited national data available that focuses on the prevalence of mental illness in Aboriginal youth, there is some data that shows that the inequality between Aboriginal and non-Aboriginal Australians’ mental health is evident from an early age. For example, in 2008 Aboriginal young people were hospitalised for mental and behavioural disorders at 1.8 times the non-Aboriginal rate [27]. The Western Australian Aboriginal Child Health Survey (WAACHS) measured a young person’s risk of emotional and behavioural difficulties by administering the Strengths and Difficulties Questionnaire. It found that for Aboriginal youth aged 12–17 years, 21% were at high risk of clinically significant emotional or behavioural difficulties, compared with 13% of non-Aboriginal youth from the same age group [37]. Despite this higher prevalence, only a small proportion (11%) of Aboriginal youth aged 12–17 years have had contact with mental health services [37]. This may be due to many factors, including remoteness, language barriers, affordability and cultural sensitivity issues [38]. Families also avoid accessing mental health services for young people, for fear of government involvement and the possible removal of children [39].

Young Aboriginal Australians are disproportionately exposed to risk factors, such as grief, trauma, loss, and discrimination which greatly affects their social and emotional wellbeing [37]. Grief and loss due to separations in past generations, as well as relating to loss of land and culture, continue to impact on the young generations of Aboriginal people [40]. Aboriginal youth are also directly impacted by the very high death rates amongst adults and suicide amongst young people [40].

The Australian Institute of Health and Welfare reports that during the period of 2005–06 the intentional self-harm hospitalisation rate was almost twice as high among Aboriginal young people compared with other young Australians in the same region [41]. Additionally, between the years of 2003–05 the age-standardised suicide rate for 12–24 year olds was more than 4 times as high among Aboriginal young people compared with non-Aboriginal young people in Queensland, Western Australia, South Australia and Northern Territory [41].

### Producing culturally appropriate MHFA guidelines for helping adolescents

The specific impetus for this research came from feedback from Aboriginal MHFA instructors attending the annual MHFA instructor conference in 2011. The feedback from instructors was that there is a great need for MHFA training on how to assist Aboriginal adolescents. Other calls have been made to increase the current levels of engagement of Aboriginal youth with mental health services by embedding elements of cultural competence [16].

Cultural differences must be considered when developing MHFA guidelines for Aboriginal adolescents. Cultural considerations might include how an Aboriginal adolescent conceptualises their world, including mental health, family, relationships and identity [42]. Symptoms of mental illness need to be understood within a cultural context [18]. Furthermore, how to best engage and communicate mental health messages with Aboriginal adolescents is a key consideration [16,43]. This is especially important in light of recent research that found there are many barriers to help-seeking amongst Aboriginal Australians, including shame, fear and low mental health literacy [44]. For these reasons it is important that MHFA guidelines are designed specifically for assisting young Aboriginal people.

This paper describes the development of expert consensus guidelines for how to provide culturally appropriate mental health first aid to Aboriginal adolescents. Given that guidelines already exist for how to assist Aboriginal people who are developing mental health problems or in mental health crisis situations, these new guidelines are focused on tailoring that assistance to the adolescent age group. They deal with cultural considerations and communication techniques in providing mental health first aid rather than specific first aid actions. The guidelines are designed to be particularly appropriate for non-Aboriginal people who are providing mental health first aid or for Aboriginal people who are from a different cultural background to the adolescent they are assisting.

## Methods

### The Delphi method

Expert consensus methods provide a systematic way of drawing on the expertise of people working in a particular area and give guidance that is readily applicable to a particular context [45]. The Delphi method involves a panel of experts making private, independent ratings of agreement with a series of statements [46]. Statements about cultural considerations and communication strategies for assisting an Aboriginal adolescent experiencing a mental health problem were derived from a search of the lay and scientific literature. These statements were presented, via online questionnaires, to a panel of experts in three sequential rounds. New strategies suggested by panel members in Round 1 were included as statements in Round 2 for all experts to rate. A summary of group ratings was fed back to the panel members after both Round 1 and Round 2. Panel members could choose to either change or maintain their original ratings, in light of the group ratings. This process resulted in a list of statements that had substantial consensus in ratings, and statements with low or conflicting ratings were discarded.

### Panel formation

The research involved the recruitment of a panel of experts in the field of Aboriginal and Torres Strait Islander youth mental health. A panel size of 23 has been found to yield stable results in a simulation study [47]. In the current study, 41 participants completed Round 1. Participants were required to identify as an Aboriginal or Torres Strait Islander person and have expertise in Aboriginal youth mental health. Potential participants were considered to have sufficient expertise if they had authored materials on Aboriginal mental health (for instance teaching notes, journal articles, books or information leaflets), had attended and presented at meetings, conferences or training in Aboriginal mental health, or were known as respected professionals through the authors’ networks. Potential participants were recruited by distributing information about the study via face-to-face meeting, telephone call and/or email, and were sent a Participant Information Sheet prior to participation. Informed consent was implied by responding to online questionnaires. Participants were reimbursed for their time (AU$250) upon completion of the final survey.

### Questionnaire development and administration

A systematic search was carried out on websites, online forums, information brochures, books, carers’ manuals, and journal articles for any written information about how to assist an Aboriginal adolescent developing a mental illness or experiencing a mental health crisis. This included a search for existing information on tips for adults on how to communicate with Aboriginal adolescents in general, as well as on mental health and other sensitive issues.

There were three major sources of information. The first was a web-based search, which involved entering *a priori* key terms into online search engines (Google Australia, Google U.K. and Google U.S.) and following the links to the first 50 sites listed for each search. The searches included various combinations of search terms, for example, *Aboriginal* AND *adolescent* AND *mental health problem*. Any links appearing on these websites, which the authors thought may contain useful information, were followed. Sources recommended by relevant mental health websites (such as Australian Indigenous *HealthInfoNet)* were also searched. The second was the Australian Institute of Aboriginal and Torres Strait Islander Studies Audiovisual Archives and Library, which was used to identify key print texts located within Australian libraries (e.g. [43]). The third was an academic journal database search (including PubMed and PsycInfo), which presented relevant clinical research and literature reviews pertaining to the topic of interest (e.g. [48,49]).

The majority of information came from websites and journal articles, as few books focused on pre-clinical interventions. We obtained suggestions for first aid strategies from approximately 11 websites, 2 books and 20 journal articles. In addition, to these new items, other statements were incorporated into the questionnaire from two previously conducted Delphi studies, one on communicating with adolescents about sensitive issues and the other on cultural considerations and communication techniques with Aboriginal adults. The conduct of these studies is described in detail elsewhere [50,14], so will not be elaborated on here, except to say that the pre-existing youth communication questionnaire items and cultural consideration items were presented to the current Aboriginal expert panel for consideration to ensure that any gaps in the Aboriginal adolescent-specific literature were still considered by the panel.

The information gathered from these sources was analysed by one of the authors (KJC) and written up as individual survey items. This document was presented to a working group consisting of the authors, who are experts in the Delphi method or Aboriginal mental health. The working group screened the items to ensure they fitted the scope of the project, were comprehensible, and had a consistent format, while remaining as faithful as possible to the original meaning of the information. After several draft surveys, the group produced a list of 304 items that formed the first survey sent to panel members.

The Round 1 survey was organised into 8 sections. Panel members rated each statement according to how important it was that the item be included in the guidelines. Statements were rated using a 5-point rating scale that included the following options: *Essential*, *Important, Don’t know/depends, Unimportant* and *Should not be included.* The Round 1 survey also included text boxes that allowed panel members provide comments or make suggestions after each page. To analyse this feedback, one of the authors (KJC) read through all the comments and wrote them up as draft first aid strategies. The working group evaluated the suggested draft strategies to determine whether they were original ideas that had not been included in the Round 1 questionnaire. Any strategy that was judged by the group to be an original idea was included as a new item to be rated in the Round 2 questionnaire.

Panel members completed the questionnaires online using SurveyMonkey [51]. This research was approved by the Australian Government Department of Health and Ageing Ethics Committee.

### Statistical analysis

On completion of each round, the survey responses were analysed by obtaining percentages for endorsement of each survey item. The following cut-off points were used:

#### Criteria for accepting an item

● If at least 90% of the panel rated an item as *Essential* or *Important* as a first aid guideline for helping an Aboriginal or Torres Strait Islander adolescent, it was included in the guidelines.

#### Criteria for re-rating an item

● If 80-89% of panel members rated an item as *Essential* or *Important*, we asked panel members to rerate that item in the next round.

● Items were re-rated once only. If an item was not endorsed after two rounds it was excluded from the guidelines.

#### Criteria for rejecting an item

● Any items that did not meet the above conditions were excluded.

Once analysis was complete, panel members were sent a report, which outlined the results of that round’s survey. The report consisted of a list of statements that had been endorsed, a list of statements that had been rejected, and a list of statements that were to be re-rated in the next survey round. The statements to be re-rated were displayed with the group percentages for each possible rating, and also with the panel member’s individual rating, so that panel members could compare their response to that of the group. Presentation of the report in this way allowed the panel members to decide whether to maintain or modify their ratings in the next survey round.

The same criteria for endorsing, excluding and re-rating statements were applied to the data collected in Round 2, with one exception. If a statement was re-rated in Round 2 and again failed to achieve a consensus of between 90 and 100 per cent across the panel, it was then excluded. Only those statements that had been entered as new statements in Round 2, and afterward fell into the re-rate category, were entered into the Round 3 survey.

### Guidelines development

All statements endorsed as either *Essential* or *Important* by ≥ 90% of panel members were written into a guideline document. One author (KJC) drafted the guidelines by writing the list of endorsed statements into sections of prose based on common themes. Where possible, statements were combined and repetition deleted to reduce length. Some items were given examples and explanatory notes to clarify the general nature of the advice, for instance, including information on the concept of ‘shame’ in Aboriginal and Torres Strait Islander communities. The draft was then presented to the working group, who edited the document to create a set of guidelines that were written in plain English and were easy to follow. A copy of the final draft was sent to panel members for review.

### Feedback from panel members

To assess the panel members’ satisfaction with the research method, participants were invited to provide their feedback after the final survey. Respondents were asked to comment on the appropriateness of the time commitment and the remuneration, whether participating was worthwhile and enjoyable, whether they believed the guidelines would be of benefit to Aboriginal people and whether they recommend the Delphi method for other research projects with Aboriginal people. Participants were asked to respond using the following 5-point scale: *Strongly agree, Agree, Neither agree nor Disagree, Disagree,* and *Strongly disagree.*

## Results

### Expert panel members

48 panel members were recruited and a total of 41 participants (27 female and 14 male) completed the Round 1 survey. Panel members were recruited from around Australia including: the Australian Capital Territory (n = 3), New South Wales (n = 11), the Northern Territory (n = 3), Queensland (n = 7), South Australia (n = 3), Victoria (n = 8) and Western Australia (n = 6). Tasmania was the only state without representation on the panel. Having a geographical spread of panel members was thought to be important to represent different experiences and attitudes of Aboriginal communities across Australia. It is therefore important to note that no participants identified as a Torres Strait Islander person. The 2011 Australian Census found that of the 548,370 Australians who identified as Indigenous, 90% were of Aboriginal descent only, 6% were of Torres Strait Islander descent only and 4% identified as being of both Aboriginal and Torres Strait Islander descent [23]. Given that Torres Strait Islander people form such a small percentage of the Indigenous population, it is unfortunate, but not surprising that it was difficult to recruit a mental health expert from this community.

Participants were experienced in a wide range of different services across the mental health field, including: private psychology clinics, counselling services, Aboriginal medical services, universities, government health services, social services, drug and alcohol treatment centres and justice services.

The panel had an average of 10.5 years experience in youth mental health (5 years or less = 22.0%, 6–10 years = 34.1%, 11–15 years = 29.3%, 16–20 years = 12.2%, more than 21 years = 2.4%).

There was a high retention rate across the surveys with 37 participants (90% of the Round 1 panel) going on to complete both Round 2 and Round 3.

### Endorsed statements

See Additional file 1 for an overview of the numbers of items that were included, excluded, created and re-rated in each round of the survey. Across the three rounds, 194 statements were rated as *Essential* or *Important* by ≥90% of the panel members (see Additional file 2). Items that were not rated as *Essential* or *Important* by at least 90% of the panel were excluded from the guidelines (see Additional file 3).

The final guidelines (see Additional file 4) provide information on how to provide mental health first aid to an Aboriginal or Torres Strait Islander adolescent in a culturally sensitive way. They provide guidance on how to make the approach, communicate effectively, support them, discuss mental health problems and assist them to find professional help and other supports (see Table 1 for the structure of the guidelines).

**Table 1:** Structure of the final guidelines

**Section**	**Topics covered**
Understanding cultural influences	Be aware of the impacts of culture and history
	Learn about the adolescent’s cultural beliefs and concept of mental illness
	Be aware of challenges the adolescent might be experiencing
	Think about the impact that family may have on the adolescent
	Understand what might cause the adolescent to feel shame
Making the approach	Approach the adolescent in a sensitive and appropriate manner
	Know how to handle concerns about cultural or gender differences
	Ask the adolescent who they wish to involve in discussions
	If you can’t help, ensure someone else does
	Engage the adolescent before discussing personal issues
Tips for good communication	Make the adolescent the focus of the interaction
	Be warm and non-judgmental
	Be honest, reliable and consistent
	Adapt your communication style
	Talk to the adolescent in a calm and confident manner
	Use clear and simple language
	Let the adolescent tell their story
	Be aware of body language
	Provide comfortable and appropriate amount of personal space
Discussing mental illness with the adolescent	Let the adolescent tell you about their experiences and beliefs
	Eating disorders in Aboriginal adolescents
	Know how to share your own experience of mental illness
Discussing options and getting help	Offer the adolescent options and assistance in finding a solution to their problem
	Recommend that the adolescent talk to a professional as soon as possible
	Encourage the use of other supports in the adolescent’s community
	In a crisis
Handling difficulties in the interaction	
Exercise self-care	

### Panel member feedback

At the end of the Round 3, survey participants were asked to give their feedback on their experiences as a participant and their views on the research methodology (see Table 2 for these results). A notable result was that in response to the statement, *I thought participating in this research was worthwhile,* all participants responded with either Strongly agree or Agree. Additionally, the majority of the participants (73%) either strongly agreed or agreed with the statement *I would recommend the Delphi method for other research projects with Aboriginal people.*

**Table 2:** Results of panel member feedback

**Item**	**Strongly agree (%)**	**Agree (%)**	**Neither agree nor disagree (%)**	**Disagree (%)**	**Strongly disagree (%)**
***I thought participating in this research was worthwhile.***	81	19	0	0	0
***I enjoyed participating in this research.***	57	41	3	0	0
***I thought the remuneration was appropriate.***	24	70	5	0	0
***I thought the time commitment was appropriate.***	27	60	14	0	0
***I would recommend the Delphi method for other research projects with Aboriginal people.***	35	38	24	3	0

## Discussion

By using experts in the field, this research aimed to produce guidelines for providing culturally appropriate mental health first aid to an Australian Aboriginal or Torres Strait Islander adolescent. The expert panel reached consensus on a range of first aid techniques, from how to initially engage the adolescent to discussing mental health problems with them. There were a number of notable findings.

### Understanding culture when providing help

The open-ended responses provided in Round 1 highlighted that culture is not static and will vary between regions, families and individuals. The findings make clear that being culturally appropriate when providing first aid includes recognising that views on mental health differ between communities. Panellists stated that the first aider should avoid making assumptions because of the adolescent’s culture (e.g. automatically assuming that unusual or out-of-character behaviours are related to culture or adopting communication styles based on cultural stereotypes). Endorsed statements also reflected the problem of relying on cultural assumptions, for example, when helping the adolescent get professional help:and

The first aider should be aware that the adolescent may not be comfortable using Aboriginal-controlled health services because of concerns about confidentiality and ‘shame jobs’ if it involves a personal problem.

The first aider should be aware of the way shame affects the behaviour of Aboriginal people; for instance, some Aboriginal people may be afraid of attending a mainstream hospital because, historically, being admitted to a hospital with a mental illness caused shame on family and community.

The open-ended responses indicated that although cultural knowledge is important when providing mental health first aid to an Aboriginal adolescent, any help is better than none, especially in a crisis situation. One participant’s comments read:

A first aider ideally will have some knowledge of the Aboriginal culture before stepping in and offering first aid to an Aboriginal person, however, this should not be a barrier to supporting a young person. Never be afraid to ask a young person to clarify or learn what you don’t know about them or their culture. All individuals, including young persons, are the experts on their own lives and culture.

Comments like this were similar to feedback in a past Delphi study with Aboriginal people where panel members suggested that a person providing first aid to an Aboriginal adult need not be so focused on cultural awareness that they lose sight of the physical and emotional needs of the person they are assisting [14]. Panellists in the current study were adamant that although it is important to be sensitive to cultural differences when having a discussion, individual differences should also be considered in the first aider’s approach (e.g. adapting communication with the adolescent to their age, level of maturity and favoured style rather than relying on any preconceptions based on culture). This meant that the present study was consistent with past findings that showed the importance of providing culturally appropriate first aid whilst having equal consideration for the individual needs of the person, regardless of their cultural identity [14].

The findings indicated that when providing help, the first aider should consider the other challenges that an Aboriginal adolescent may be facing, for example, social problems due to racism or discrimination, many deaths or losses of family or friends or anger from past injustices. A caveat to this idea was that the first aider should not assume that an adolescent is facing particular problems, for example one participant noted:

It is important to recognise historical factors that may lead to shame but essential that the first aider takes the adolescent on face value without pushing previous trauma upon them.

### Importance of autonomy

A strong theme throughout the panel feedback was the importance of giving the adolescent autonomy and choice. For example, panel members discussed the need for the first aider to ask the adolescent before involving family or community members, giving the adolescent the opportunity to answer questions even if their family members are present, empowering the adolescent to make decisions, and allowing the adolescent to drive the discussion.

### Similarities with a previous study on helping an adolescent

There were several commonalities between items endorsed in the present study and those endorsed in a previous study on communicating with Australian adolescents about sensitive issues [50]. There were items that were endorsed in both studies, for example, asking the adolescent where they feel comfortable and safe to talk, taking the time to build rapport and trust, being reliable and consistent, listening without interrupting, being genuine, talking calmly, awareness of body language, how to discuss problems and offering courses of action. These commonalities show that Aboriginal adolescents have much in common with other Australian adolescents, even though there are specific considerations to take into account as well.

### Panel member feedback

Panel member feedback at the end of Round 3 was similar to that of a past Delphi study with Aboriginal experts to develop mental health first aid guidelines [14]. For example, in both the present study and the previous study, all panel members responded with either *Strongly agree* or *Agree* to the statement, *I thought participating in this research was worthwhile.* Statements regarding the appropriateness of the Delphi research method also received a high level of agreement, with 73% of participants in the present study responding with either *Strongly agree* or *Agree* to the statement *I would recommend the Delphi method for other research projects for Aboriginal people.* This was similar to the 83% of participants in the previous study responding with either *Strongly agree* or *Agree* to that same statement.

When given the opportunity to provide feedback on the research project, participants gave reasons why they liked the methodology, including that they appreciated the community consultation and that they wished more organisations would take such an approach. Participants also stated that the combination of multiple surveys and the results report, provided to them after each round, ensured all the necessary content was considered. Participants also made suggestions for future studies, for example that consumers, parents, elders and both Aboriginal and non-Aboriginal mental health experts might be included as participants. Another common response was a justification of why *Don’t know/depends* was selected for many survey statements. This included the acknowledgement that often the mental health first aid strategies used will depend on the individual adolescent’s personality, geographical area, severity of illness, access to services and the availability of other supports.

### Dissemination of guidelines

To have a direct impact on Aboriginal adolescents and their communities, the developed guidelines must be disseminated throughout Australia. The guidelines will inform a Youth Aboriginal and Torres Strait Islander MHFA Supplementary Booklet, which will supplement both the Aboriginal and Torres Strait Islander MHFA training program and the Youth MHFA training program.

Mental Health First Aid courses are widely disseminated in Australia, with over 1,000 instructors offering courses and over 200,000 people having received the training, which is more than 1% of the adult population [5]. In addition, there are 164 Aboriginal and Torres Strait Islander instructors and over 13,500 people have completed the Aboriginal and Torres Strait Islander MHFA course. Thus, there is a ready means of national dissemination for the newly developed guidelines, supplementary workbook and updated courses.

In addition, the guidelines will be available from the Mental Health First Aid website [52] and will be submitted to the NHMRC Clinical Practice Guidelines Portal [53]. Copies of MHFA guidelines are downloaded very frequently from the Mental Health First Aid website. The guidelines web page has attracted an average of 45 visits per day. A recent evaluation found that the information in the guidelines led to more positive, empathic and successful helping behaviours [54]. These findings suggest that the newly developed guidelines might also be practically useful when made available online.

The guidelines received provisional support from the experts involved in this study. Despite this, and the widespread dissemination plan outlined, only further evaluation of the first aid outcomes will confirm whether or not the information developed by this research is effective in increasing support provided to Aboriginal Australian youth with mental health problems.

## Conclusions

Aboriginal and Torres Strait Islander Youth mental health experts were able to reach consensus about what the appropriate communication strategies for providing mental health first aid to an Aboriginal and Torres Strait Islander adolescent. The information in these guidelines will be made available to communities throughout Australia. The findings of this study will also be used to create a supplementary booklet and inform future MHFA training programs. These outcomes will help ensure that the community provides the best possible support to Aboriginal adolescents who are developing mental illnesses or are in a mental health crisis.

## Endnote

^a^*Aboriginal* in this article refers to Aboriginal and Torres Strait Islander people.

## Abbreviations

MHFA: Mental health first aid; WAACHS: Western Australian Aboriginal Child Health Survey.

## Competing interests

The authors declare that they have no competing interests.

## Authors’ contributions

KJC carried out the systematic literature search, was involved in panel member recruitment, drafted the surveys, carried out the data collection and analysis, drafted the guidelines, and drafted the manuscript. AFJ and CMK participated in the conception and design of the Delphi research protocol, participated in the working group and helped with the drafting of the manuscript. BAK participated in the conception and design of the Delphi research protocol and participated in the working group. KSB participated in the working group. AJW recruited expert panel members and participated in the working group. All authors read and approved the final manuscript.

## Supplementary Material

Additional file 1 Overview of items included, excluded, created and re-rated in each round of the survey. Click here for file

Additional file 2 Items that received ≥90% consensus from the expert panel and were accepted in guidelines. Click here for file

Additional file 3 Items excluded from the guidelines. Click here for file

Additional file 4 Communicating with an Aboriginal or Torres Strait Islander Adolescent: Guidelines for being culturally appropriate when providing mental health first aid. Click here for file

## References

[B1] Slade T Johnston A Oakley Browne MA Andrews G Whiteford H National survey of mental health and wellbeing: methods and key findings Aust N Z J Psychiatry 2007 2009 43 594 605 10.1080/00048670902970882 19530016

[B2] Reavley NJ Jorm AF Public recognition of mental disorders and beliefs about treatment: changes in Australia over 16 years Br J Psychiatry 2012 200 419 425 10.1192/bjp.bp.111.104208 22442098

[B3] Jorm AF Korten AE Jacomb PA Rodgers B Pollitt P Beliefs about the helpfulness of interventions for mental disorders: a comparison of general practitioners, psychiatrists and clinical psychologists Aust N Z J Psychiatry 1997 31 844 851 10.3109/00048679709065510 9483257

[B4] Kitchener BA Jorm AF Mental health first aid: an international programme for early intervention Early Interv Psychiatry 2008 2 55 61 10.1111/j.1751-7893.2007.00056.x 21352133

[B5] Jorm AF Kitchener BA Noting a landmark achievement: mental health first aid training reaches 1% of Australian adults Aust N Z J Psychiat 2011 45 808 813 10.3109/00048674.2011.594785 21827342

[B6] Kelly CM Mithen JM Fischer JA Kitchener BA Jorm AF Lowe A Scanlan C Youth mental health first aid: a description of the program and an initial evaluation Int J Ment Health Syst 2011 5 4 10.1186/1752-4458-5-4 21272345 PMC3041764

[B7] Kelly CM Jorm AF Kitchener BA Langlands RL Development of mental health first aid guidelines for suicidal ideation and behaviour: a Delphi study BMC psychiatry 2008 8 17 10.1186/1471-244X-8-17 18366657 PMC2324091

[B8] Langlands RL Jorm AF Kelly CM Kitchener BA First aid recommendations for psychosis: using the Delphi method to gain consensus between mental health consumers, carers, and clinicians Schizophr Bull 2008 34 435 443 17768307 10.1093/schbul/sbm099 PMC2632434

[B9] Langlands RL Jorm AF Kelly CM Kitchener BA First aid for depression: a Delphi consensus study with consumers, carers and clinicians J Affect Disord 2008 105 157 165 10.1016/j.jad.2007.05.004 17574684

[B10] Kingston AH Jorm AF Kitchener BA Hides L Kelly CM Morgan AJ Hart LM Lubman DI Helping someone with problem drinking: mental health first aid guidelines - a Delphi expert consensus study BMC Psychiatry 2009 9 79 10.1186/1471-244X-9-79 19968868 PMC2799400

[B11] Hart LM Jorm AF Paxton SJ Kelly CM Kitchener BA First aid for eating disorders Eat Disord 2009 17 357 384 10.1080/10640260903210156

[B12] Kelly CM Jorm AF Kitchener BA Development of mental health first aid guidelines on how a member of the public can support a person affected by a traumatic event: a Delphi study BMC Psychiatry 2010 10 49 10.1186/1471-244X-10-49 20565918 PMC2904289

[B13] Kingston AH Morgan AJ Jorm AF Hall K Hart LM Kelly CM Lubman DI Helping someone with problem drug use: a Delphi consensus study of consumers, carers, and clinicians BMC psychiatry 2011 11 3 10.1186/1471-244X-11-3 21208412 PMC3022807

[B14] Hart LM Jorm AF Kanowski LG Kelly CM Langlands RL Mental health first aid for indigenous Australians: using Delphi consensus studies to develop guidelines for culturally appropriate responses to mental health problems BMC Psychiatry 2009 9 47 10.1186/1471-244X-9-47 19646284 PMC2729076

[B15] Vicary DA Bishop BJ Western psychotherapeutic practice: engaging Aboriginal people in culturally appropriate and respectful ways Aust Psychologist 2005 40 8 19 10.1080/00050060512331317210

[B16] Westerman T Engaging Australian Aboriginal youth in mental health services Aust Psychologist 2010 45 212 222 10.1080/00050060903451790

[B17] Isaacs AN Pyett P Oakley-Browne MA Gruis H Waples-Crowe P Barriers and facilitators to the utilization of adult mental health services by Australia’s Indigenous people: seeking a way forward Int J Ment Health Nurs 2010 19 75 82 10.1111/j.1447-0349.2009.00647.x 20367644

[B18] Parker R Australia’s aboriginal population and mental health J Nerv Ment Dis 2010 198 3 7 10.1097/NMD.0b013e3181c7e7bc 20061862

[B19] Johnstone MJ Kanitsaki O An exploration of the notion and nature of the construct of cultural safety and its applicability to the Australian health care context J Transcult Nurs 2007 18 247 256 10.1177/1043659607301304 17607062

[B20] Whaley AL Longoria RA Assessing cultural competence readiness in community mental health centers: a multidimensional scaling analysis Psychol Serv 2008 5 169 183

[B21] Mathieson F Mihaere K Collings S Dowell A Stanley J Maori cultural adaptation of a brief mental health intervention in primary care J Prim Health Care 2012 4 231 238 22946072

[B22] Durey A Thompson SC Reducing the health disparities of Indigenous Australians: time to change focus BMC Health Serv Res 2012 12 151 10.1186/1472-6963-12-151 22682494 PMC3431273

[B23] Australian Bureau of Statistics 2075.0 - Census of population and housing - counts of Aboriginal and Torres Strait Islander Australians, 2011 http://www.abs.gov.au/ausstats/abs@.nsf/mf/2075.0

[B24] Vos T Barker B Begg S Stanley L Lopez AD Burden of disease and injury in Aboriginal and Torres Strait Islander peoples: the Indigenous health gap Int J Epidemiol 2009 38 470 477 10.1093/ije/dyn240 19047078

[B25] Jorm AF Bourchier SJ Cvetkovski S Stewart G Mental health of Indigenous Australians: a review of findings from community surveys Med J Aust 2012 196 118 121 10.5694/mja11.10041 22304605

[B26] Australian Bureau of Statistics 4704.0 - The health and welfare of Australia’s Aboriginal and Torres Strait Islander peoples, oct 2010 http://www.abs.gov.au/AUSSTATS/abs@.nsf/lookup/4704.0Chapter420Oct+2010

[B27] Australian Institute of Health and Welfare The health and welfare of Australia’s Aboriginal and Torres Strait Islander people, an overview 2011 http://www.aihw.gov.au/WorkArea/DownloadAsset.aspx?id=10737418955

[B28] Australian Bureau of Statistics 3309.0 - suicides, Australia, 2010 http://abs.gov.au/AUSSTATS/abs@.nsf/mf/3309.0/

[B29] Swan P Raphael B Ways forward: national Aboriginal and Torres Strait Islander mental health policy; national consultancy report, 1995 http://www.health.gov.au/internet/publications/publishing.nsf/Content/mental-pubs-w-wayforw-toc

[B30] Kanowski LG Jorm AE Hart LM A mental health first aid training program for Australian Aboriginal and Torres Strait Islander peoples: description and initial evaluation Int J Ment Health Syst 2009 3 10 10.1186/1752-4458-3-10 19490648 PMC2694763

[B31] Vicary D Westerman T ‘That’s just the way he is’: some implications of Aboriginal mental health beliefs Adv in Mental Health 2004 3 103 112 10.5172/jamh.3.3.103

[B32] Day A Francisco A Social and emotional wellbeing in Indigenous Australians: identifying promising interventions Aust N Z J Public Health 2013 37 350 355 10.1111/1753-6405.12083 23895478

[B33] Kieling C Baker-Henningham H Belfer M Conti G Ertem I Omigbodun O Rohde LA Srinath S Ulkuer N Rahman A Child and adolescent mental health worldwide: evidence for action Lancet 2011 378 1515 1525 10.1016/S0140-6736(11)60827-1 22008427

[B34] Oakley Browne MA Wells JE Scott KM McGee MA Lifetime prevalence and projected lifetime risk of DSM-IV disorders in Te Rau Hinengaro: the New Zealand mental health survey Aust N Z J Psychiatry 2006 40 865 874 10.1080/j.1440-1614.2006.01905.x 16959012

[B35] Kelly CM Kitchener BA Jorm AF Youth mental health first aid: a manual for adults assisting young people 2013 3 Melbourne, Australia: Mental Health First Aid Australia

[B36] Australian Bureau of Statistics 1301.0 - Year book Australia, 2012 http://www.abs.gov.au/ausstats/abs@.nsf/mf/1301.0

[B37] Zubrick S Silburn S Lawrence D Mitrou F Dalby R Blair E Griffin J Milroy H De Maio J Cox A Li J The western Australian Aboriginal child health survey: the social and emotional wellbeing of Aboriginal children and young peoplehttp://aboriginal.childhealthresearch.org.au/kulunga-research-network/waachs.aspx

[B38] Butler C Indigenous adolescent mental health: what is the role of primary health care? RESEARCH ROUNDup 2012 24 n.p

[B39] Williamson AB Raphael B Redman S Daniels D Eades SJ Mayers N Emerging themes in Aboriginal child and adolescent mental health: findings from a qualitative study in Sydney, New South Wales Med J Aust 2010 192 603 605 20477741 10.5694/j.1326-5377.2010.tb03649.x

[B40] Dobia B O’Rourke VG Promoting the mental health and wellbeing of Indigenous children in Australian primary schoolshttp://www.kidsmatter.edu.au/sites/default/files/public/promoting-mental-health-wellbeing-indigenous-children.pdf

[B41] Eldridge D Australian Institute of Health and Welfare Injury among young Australianshttp://www.aihw.gov.au/publication-detail/?id=6442468094

[B42] Williamson AB Raphael B Redman S Daniels J Eades SJ Mayers N Emerging themes in Aboriginal child and adolescent mental health: findings from a qualitative study in Sydney, New South Wales Med J Aust 2010 192 603 605 20477741 10.5694/j.1326-5377.2010.tb03649.x

[B43] Reed-Gilbert K Brown S Our place: stories about good practice in youth work with Aboriginal young people 2002 South Sydney Youth Services: Sydney

[B44] Price M Dalgleish J Help-seeking among indigenous Australian adolescents: exploring attitudes, behaviours and barriers Youth Studies Australia 2013 31 10 18

[B45] Minas H Jorm AF Where there is no evidence: use of expert consensus methods to fill the evidence gap in low-income countries and cultural minorities Int J Ment Health Syst 2010 4 33 10.1186/1752-4458-4-33 21176157 PMC3016371

[B46] Jones J Hunter D Consensus methods for medical and health services research BMJ 1995 311 376 380 10.1136/bmj.311.7001.376 7640549 PMC2550437

[B47] Akins RB Tolson H Cole BR Stability of response characteristics of a Delphi panel: application of bootstrap data expansion BMC Med Res Methodol 2005 5 37 10.1186/1471-2288-5-37 16321161 PMC1318466

[B48] O’Brien A Factors shaping Indigenous mental health: an ethnographic account of growing up Koori from a Gubba perspective Aust J Holist Nurs 2005 12 11 12 19175266

[B49] McNamara PM Adolescent suicide in Australia: rates, risk and resilience Clin Child Psychol Psychiatry 2012 18 351 369 23118313 10.1177/1359104512455812

[B50] Fischer JA Kelly CM Kitchener BA Jorm AF Development of guidelines for adults on how to communicate with adolescents about mental health problems and other sensitive topics: a Delphi study SAGE Open 2013 3 4 DOI: 10.1177/2158244013516769

[B51] SurveyMonkey [http://www.surveymonkey.com]

[B52] Mental Health First Aid Australia [http://www.mhfa.com.au]

[B53] NHMRC Clinical Practice Guidelines Portal [http://www.clinicalguidelines.gov.au]

[B54] Hart LM Jorm AF Paxton SJ Cvetkovski S Mental health first aid guidelines: an evaluation of impact following download from the world wide web Early Interv Psychiatry 2012 6 399 406 10.1111/j.1751-7893.2012.00345.x 22379952

