# Supplementary figures and images for: Providing culturally appropriate mental health first aid to an Aboriginal or Torres Strait Islander adolescent: development of expert consensus guidelines
Source: Int J Ment Health Syst. 2014 Jan 28;8:6. doi: 10.1186/1752-4458-8-6 (PMC3922159; doi:10.1186/1752-4458-8-6)

**Overview of items included, excluded, created and re-rated in each round of the survey.**

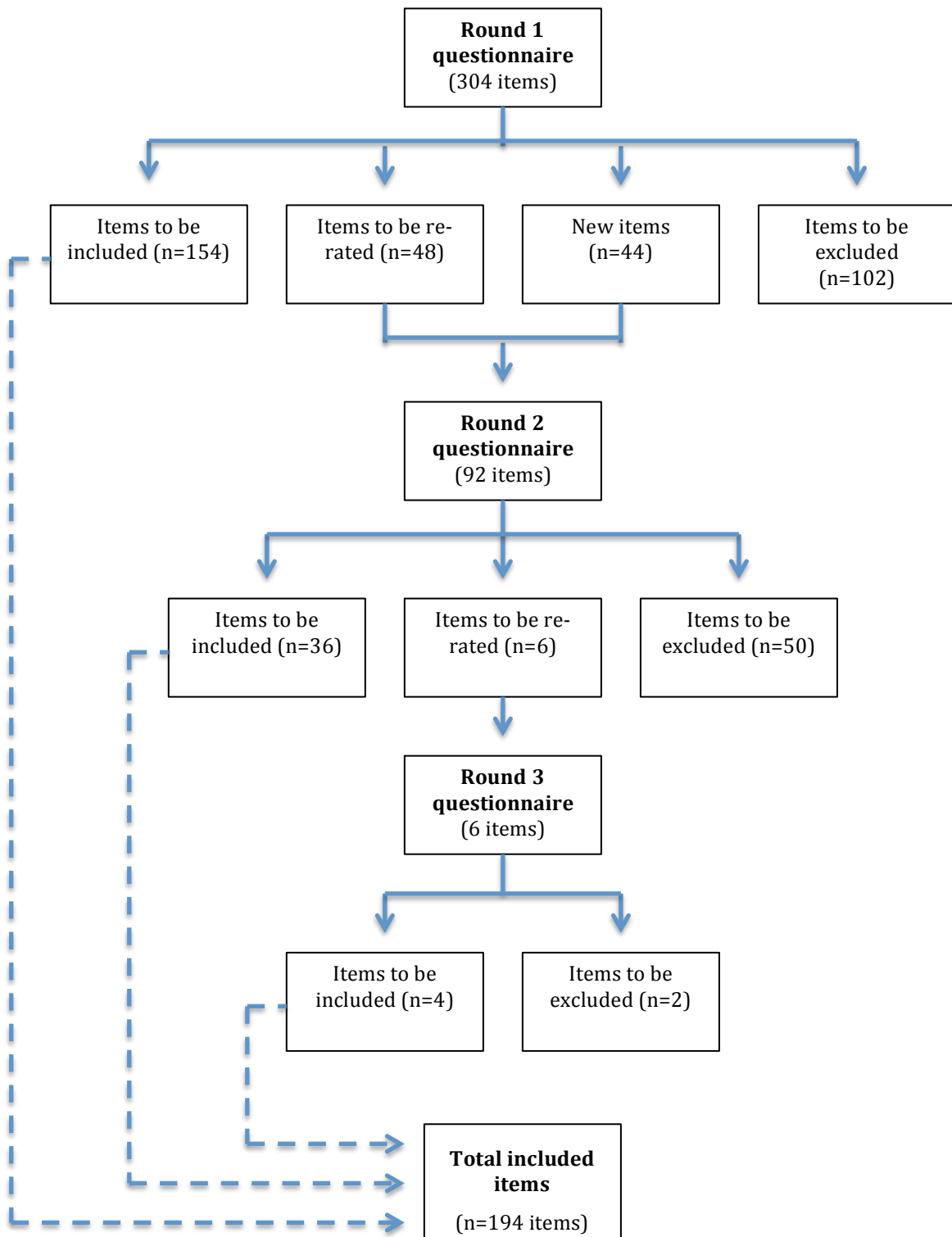

Supplement: Additional file 1 — Overview of items included, excluded, created and re-rated in each round of the survey. [file 1752-4458-8-6-S1.pdf]
