# Supplementary material for: Providing culturally appropriate mental health first aid to an Aboriginal or Torres Strait Islander adolescent: development of expert consensus guidelines
Source: Int J Ment Health Syst. 2014 Jan 28;8:6. doi: 10.1186/1752-4458-8-6 (PMC3922159; doi:10.1186/1752-4458-8-6)
Supplement: Additional file 2 — Items that received ≥90% consensus from the expert panel and were accepted in guidelines. [file 1752-4458-8-6-S2.pdf]

Items that received ≥90% consensus from the expert panel and were accepted in guidelines

**Understanding cultural influences**	**Round**
The first aider should have a basic awareness of the historical invasion of Australia by Europeans and the subsequent impact on the health and wellbeing of Aboriginal people.	1
When approaching the adolescent, the first aider should be aware of the adolescent's cultural background, local cultural norms and the hierarchy of decision-making power within their community.	1
When approaching the adolescent, the first aider should be aware that there are cultural differences between Aboriginal communities.	2
If the first aider is providing mental health first aid outside their own community then they should be culturally competent and practice cultural safety.	1
The first aider should be aware that no one is ever entirely culturally competent as culture changes and evolves with time.	3
Even if the first aider does not have cultural training, they should still provide mental health first aid to the Aboriginal adolescent, because some support, especially in a crisis, is better than none.	2
*The first aider should be aware that their concepts of mental health may differ from the adolescent's.	1
The first aider should learn about the specific cultural beliefs that surround mental illness in the adolescent's community.	1
The first aider should consider that the adolescent might understand mental health within a wider context of health and wellbeing which includes concepts of social and emotional functioning.	1
The first aider should be aware of the concept of mental illness, including symptoms, behaviour and terminology used by the adolescent's community.	1

Understanding cultural influences (continued)	Round
The first aider should take into consideration the spiritual and/or cultural context of the adolescent’s behaviours.	1
The first aider should be aware that the adolescent may see behavioural signs of mental illness as part of a person’s spirit or personality and may not conceptualise them as a form of treatable mental illness.	1
The first aider should be aware that certain cultural experiences of Aboriginal people (such as seeing spirits or hearing voices of recently deceased loved ones) may be misdiagnosed or mislabelled as symptoms of mental illness.	1
The first aider should not assume that unusual or out-of-character behaviours are a part of the adolescent's culture as they may be signs of mental health problems.	2
The first aider should be aware that previous misdiagnosis of a mental health problem within the Aboriginal adolescent's community could be a barrier to help-seeking.	1
*The first aider should be aware that the adolescent may have additional challenges because of social problems such as racism and discrimination.	1
The first aider should be aware that the adolescent is likely to have experienced the death of a family or community member or friends.	1
The first aider should be aware that the adolescent may have experienced more than one death of a loved one in a short space in time so that the recovery process from each loss is very difficult.	1
The first aider should be aware of how the adolescent's family deals with death and grieving because this will vary between regions and families, e.g. some communities believe that mentioning the names of deceased relatives will call the person's spirit back.	2
The first aider should address the death of a person in a sensitive manner and be prepared to be led by the adolescent when discussing loss and any deceased relatives or friends.	2
*When discussing death and a loss experienced by the adolescent, the first aider should be aware that suppressed emotions may come to the surface.	2
The first aider should be aware that the adolescent may carry a lot of anger from injustices they or their family have endured.	1

Understanding cultural influences (continued)	Round
*If the adolescent appears angry, irritable or frustrated, the first aider should not automatically assume it is a sign of an underlying mental health problem because the adolescent might be expressing these feelings due a range of other factors, e.g. social disadvantage and racism.	2
*The first aider should recognise the importance to the adolescent of strong family ties and the cultural significance of family.	1
*The first aider should be aware that, because of the importance of strong family ties and the cultural significance of family within Aboriginal communities, problems within the family can have a greater impact on the adolescent.	1
The first aider should not criticise members of the adolescent's extended family.	1
If the family of the adolescent are present, the first aider should always ensure the adolescent has the opportunity to answer any questions, even though the family may answer for the adolescent.	2
The first aider should avoid asking the adolescent questions in front of their family or friends that might cause the adolescent embarrassment.	1
*The first aider should be aware that in some communities the stigma around mental health is strong and so the first aider needs to be very sensitive and careful when approaching an adolescent and their family who may be unfamiliar with mental health issues.	2
The first aider should be aware of the cultural concept of shame within the adolescent's community.	1
*The first aider should understand what might cause the adolescent to feel shame, such as topics or behaviours that may be considered 'mad', abnormal, unusual or embarrassing.	1
The first aider should avoid making the adolescent feel shame.	1
The first aider should not talk about the adolescent's mental health problems in front of other community member because this may result in shame.	2
The first aider should understand how an Aboriginal adolescent might feel societal shame.	1
Though the first aider should be aware of the historical factors that may lead to shame, the first aider should approach the adolescent with an open mind and be careful not to push this previous trauma upon them.	2

Making the approach	Round
The first aider should be aware that when Aboriginal people introduce themselves, they may do this in relation to their land/country, cultural background or origin.	1
If the first aider is Aboriginal they should introduce themselves in relation to their land/country, cultural background or origin.	1
The first aider should offer their first name to create a less formal atmosphere – using titles such as doctor or mister creates a hierarchy, which the adolescent may perceive as wanting power over them.	2
*The first aider should ask the adolescent where they feel comfortable or safe to talk.	1
The first aider should be aware that the adolescent is more likely to engage with them in a setting that is within the adolescent's comfort zone, e.g. in a café or at home.	1
*The first aider should make sure that they approach the adolescent privately about their experiences, at a time and place that is convenient and free of distractions.	1
When the first aider approaches the adolescent for a discussion, the first aider should do their best to ensure they will have plenty of time for the discussion.	2
The first aider should be aware that the adolescent may feel more able to discuss their problems when no one else is listening.	1
When asking the adolescent if they wish to do a mutual activity, the first aider should ensure that they inform the adolescent that it is for the purpose of having a chat (so they don't feel pressured or hemmed in), e.g. "Would you like to go for a walk with me? I want to have a chat with you."	1
The first aider should be aware that building a trusting relationship where the adolescent feels comfortable is more important than other factors (such as the place of discussion or the first aider's dress style).	1
Although some adolescents prefer confiding in first aiders of their own cultural background, don't assume that this is always the case, ask if they would prefer this.	1

Making the approach (continued)	Round
*If the adolescent shows any concern about a cultural or gender difference between themselves and the first aider, the first aider should explore the possibility of getting help from someone the adolescent feels more comfortable with.	1
The first aider should be aware that gender and cultural differences between themselves and the adolescent might be exacerbated by discussing private issues, such as commenting on the quality of family relationships, discussing intimate relationships, and, most particularly, topics that include any issues of a sexual nature.	1
The first aider should ask the adolescent if they would feel more comfortable talking away from family or friends.	1
Because family and friends are a very big part of Aboriginal culture, the first aider should anticipate that family/friends may expect to be involved in caring for the adolescent.	1
If family/friends express that they wish to be involved in caring for the adolescent, the first aider should make sure the adolescent is okay with this.	2
The first aider should allow the adolescent to choose who they talk to and who is present in these discussions.	2
If the adolescent doesn't feel comfortable talking to the first aider, the first aider should help the adolescent to find a more suitable person to talk to.	1
*If the first aider suggests that the adolescent speak to someone else about their problem, the first aider should find a suitable replacement, rather than leaving this task for the adolescent, e.g. 'I don't know if I can offer you the best advice on X, but I can help you find someone else to talk to.'	1
*If the adolescent asks the first aider for help and the first aider does not know much about the problem, the first aider should still try to support the adolescent and assist them to get other help.	1
The first aider should take the time to engage with the adolescent first (e.g. getting them to talk about their interests and social life) before discussing personal issues.	1
If the first aider doesn't know about things the adolescent is interested in, they should try to learn more from the adolescent.	2
The first aider should tell the adolescent that they don't have to talk about anything until they are ready to do so.	1

Making the approach (continued)	Round
The first aider should avoid pressuring the adolescent to talk with them.	3
*The first aider should tell the adolescent that when they want to talk, the first aider will listen to them.	1
The first aider should be aware that their reaction to the adolescent's 'everyday problems' may be important in how the adolescent decides what else to share.	1
The first aider should be aware that some adolescents (especially boys) may fear opening up about their problems in case their vulnerability is labelled as a weakness.	1
The first aider should be aware that the adolescent may hide or play down their problem if they are worried about upsetting or disappointing the first aider.	1
The first aider should not give up on trying to engage with the adolescent if the adolescent is finding it difficult to open up, but rather try again another time.	1
*The first aider should be aware that the adolescent may need more than one conversation to open up about what is bothering them.	1
*The first aider should not presume that the adolescent does not want their help even if the adolescent has an initial negative reaction to them.	1
The first aider should be aware that sometimes adolescents struggle to ask for assistance, or reject help when offered, even if they feel that a situation is out of control.	1
Once the first aider has the adolescent's trust, the first aider should tell them they want to support them.	3
The first aider should tell the adolescent that their conversation will remain private unless they talk about harming themselves or someone else.	1

Tips for good communication	Round
The first aider should be aware that the adolescent may not wish to open up until they feel that the first aider cares enough, is trustworthy and is willing to listen.	1
*The first aider should take the time to build rapport and trust with the adolescent.	1
If the first aider is non-Aboriginal they should be aware that it may take longer to build trust with the adolescent.	1
The first aider should not express any negative opinions about the adolescent's culture.	1
*If the adolescent has disengaged from other people, it is important for the first aider to allow additional time to build trust with the adolescent.	1
*The first aider should be aware that what is a respectful way to communicate with the adolescent, including body language, seating position and use of certain words, may differ between communities and regions.	1
Rather than automatically adopting communication styles based on assumptions, the first aider should notice what the adolescent feels comfortable with and use this to guide their communication.	2
*If the adolescent has difficulties with communication or literacy (e.g. vision impairment, cognitive impairment, poor literacy) the first aider should do their best to adapt their communication to meet these needs.	1
The first aider should be aware that some adolescents do not communicate well verbally, and it is important to adapt to the adolescent's preferred communication style, e.g. using art.	2
*The first aider should set aside their own issues when communicating with the adolescent and try to focus on the adolescent's own concerns.	1
The first aider should avoid stereotyping adolescents, e.g. "…teenagers always like to be difficult and oppose adults."	1
*The first aider should give the adolescent their full attention.	1
The first aider should be honest when interacting with the adolescent.	1
*The first aider should treat the adolescent with respect and fairness.	1

Tips for good communication (continued)	Round
*The first aider should recognise that each adolescent's situation and needs are unique.	1
*The first aider should be non-judgmental when speaking with the adolescent.	1
The first aider should try to empathise with how the adolescent feels.	1
The first aider should offer positive feedback to the adolescent, as this may encourage them to communicate with the first aider, e.g. "I think it's great that you are willing to talk to me about this."	3
The first aider should convey a message of hope to the adolescent by assuring them that help is available and things can get better.	1
The first aider should be caring and show warmth toward the adolescent.	1
The first aider should help the adolescent feel more positive about themselves.	1
*The first aider should be reliable and consistent in their behaviour with the adolescent.	1
The first aider should offer the adolescent consistent emotional support and understanding.	1
The first aider should acknowledge the adolescent's expertise about their own life.	1
The first aider should not contradict or minimise the adolescent's feelings by using statements such as "...you're not depressed, you're just bored."	1
*The first aider should not make any promises to the adolescent that cannot be kept.	1
The first aider should not be overly assertive or 'big note' themselves because it is not considered polite in Aboriginal society.	1
The first aider should avoid confronting the adolescent and should not criticise or blame them.	2
The first aider should note any topic the adolescent is finding distressing and give the adolescent time to think and the opportunity to continue the conversation after the pause.	2
The first aider should not criticise or blame the adolescent.	1
The first aider should avoid acting in a confrontational manner to the adolescent.	1

Tips for good communication (continued)	Round
The first aider should show a confident manner when interacting with the adolescent.	1
The first aider should avoid sounding condescending or patronising.	1
The first aider should be genuine by 'being themselves'. Adolescents are particularly tuned in to anyone who is 'faking it'.	1
The first aider should be aware that adolescents are very good at 'reading' an adult's attitude.	1
*The first aider should talk 'with', not 'at', the adolescent.	1
The first aider should use a calm voice and steady tone when speaking with the adolescent.	1
The first aider should stay calm when talking with the adolescent.	1
The first aider should never raise their voice to the adolescent if they can help it.	1
The first aider should try to be aware of when the adolescent is not listening to them and respond by changing the way they say or do things.	1
The first aider should ask the adolescent to explain their experiences rather than make their own interpretation.	1
The first aider should ask the adolescent about their experiences and how they feel about them.	1
The first aider should use simple and clear language when talking with the adolescent.	1
*If the first aider is using language that the adolescent does not understand (e.g. metaphor or humour) the first aider should change their approach and use simpler language.	2
The first aider should be aware that the adolescent may not respond to a question with a direct answer.	1
The first aider should be aware that asking for a clear response to a question of a personal nature may result in the adolescent feeling shame.	2
The first aider should allow the adolescent the opportunity to "have a yarn or tell the story" as this may work better than asking 'yes' or 'no' questions.	1
The first aider should be aware that the adolescent may not like attention being drawn to them and should keep this in mind when talking with them.	1

Tips for good communication (continued)	Round
As far as possible, the first aider should let the adolescent set the pace and style of the interaction.	2
The first aider should allow periods of silence while the adolescent considers their response to a question.	1
After speaking, the first aider should be patient and allow plenty of time for the adolescent to collect their thoughts, reflect on their feelings and decide what to say next.	1
If the first aider often uses long silent pauses, they should explain that they are just thinking about the adolescent's options and talk them through this, as the adolescent may otherwise misinterpret this negatively.	1
The first aider should listen to the adolescent without interrupting them.	1
*The first aider should actively listen and ask relevant questions to check their understanding and acknowledge that they have heard what the adolescent has said.	1
The first aider should make a conscious effort to listen, to hear the feelings and meaning behind the adolescent's words and respond to this. For example, when an adolescent says, "There's no point in going to school anymore", it could mean that they feel that the future is hopeless.	1
If a parent or guardian is present, the first aider should encourage turn-taking and courteous silence when another person is speaking.	2
If the first aider is having a private discussion with the adolescent and other people arrive, the first aider should take a moment to ask the adolescent in private what they would like to do, e.g. continue the discussion in front of others, ask others to leave or make another time to continue your discussion.	1
The first aider should notice how much personal space the adolescent feels comfortable with and not intrude beyond that.	**1**
The first aider should consider the possible ramifications of physical contact with the adolescent, as it may cause problems with personal boundaries and may lead to legal troubles.	1

Tips for good communication (continued)	Round
If the first aider represents an organisation (e.g. a teacher) they should consider their employer's guidelines on physical contact with an adolescent, before giving the adolescent a brief hug or touch of the hand.	1
The first aider should be aware that the adolescent may be uncomfortable with direct eye contact.	1
*The first aider should be aware of their own body language and what this conveys when communicating with the adolescent, e.g. posture, facial expressions and gestures.	1
The first aider should avoid negative body language such as crossing their arms, hands on hips or looking uninterested.	1
The first aider should use cues like nodding to keep a conversation going with the adolescent.	1
The first aider should avoid distracting gestures, such as fidgeting with a pen, glancing at other things or tapping their feet or fingers, as these could be interpreted as lack of interest.	2
If the first aider feels startled or disturbed by what the adolescent says, the first aider should remain neutral, e.g. phrasing, vocal tone and body language.	1
The first aider should be aware of the adolescent's body language as this can provide clues to how they are feeling or how comfortable they feel talking with the first aider.	1
If the adolescent appears defensive, the first aider should make their body language as open as possible, e.g. by appearing relaxed, sitting alongside the adolescent but angled toward them and keeping their voice calm and low.	2

**Discussing mental illness with the adolescent**	**Round**
*The first aider should be aware that the adolescent may not use mental health terms when communicating that they are feeling mentally unwell, e.g. "I feel like crap" rather than "I feel depressed" or "I'm feeling anxious".	1
*The first aider should be aware that the adolescent may hold stigmatising attitudes towards mental illness.	1
The first aider should be careful in applying labels to the adolescent that the adolescent may find stigmatising, e.g. mentally ill, drug addict.	1
The first aider should be careful not to communicate stigmatising attitudes about the adolescent's mental health problems.	1
The first aider should talk openly with the adolescent about mental illness, adapting their language to the adolescent's age and maturity.	2
When acknowledging the adolescent's illness or discomfort, the first aider should use the words the adolescent uses to describe their problems, e.g. they may say they feel 'anxious' or they may say they are 'not feeling too good'.	2
The first aider should not use scare tactics or threats when talking to the adolescent, e.g. "If you keep thinking like this, you'll end up in big trouble."	1
The first aider should never tell the adolescent to "snap out of it" or "stop thinking that way".	1
The first aider should ask the adolescent's permission before asking questions about sensitive topics, e.g. "I would like to talk about something important, but I'm aware it might be painful for you. Is that okay?"	1
*The first aider should allow the adolescent to talk to them about their experiences and beliefs about mental illness if they want to.	1
The first aider should let the adolescent speak and tell their story and then help after they have said their piece.	1
The first aider should tell the adolescent that they have done the right thing in talking to someone about their problems.	1
The first aider should not imply to the adolescent that simply talking to the first aider about their mental health problems will make these problems go away.	1
The first aider should offer options for actions that could help with the problem and allow for compromise to give the adolescent a sense of control.	1

Discussing mental illness with the adolescent (continued)	Round
The first aider should help the adolescent find solutions without trying to fix the problem for them.	1
If the first aider is concerned about the adolescent's safety or risk of harm they should ask them directly about this.	1
If the adolescent is in a potentially harmful situation (e.g. experiencing abuse or bullying), the first aider should reassure the adolescent that they want to keep them safe.	1
Although some people believe that Aboriginal adolescents are not affected by eating disorders and body dissatisfaction, the first aider should be aware that this is a myth. Therefore, the first aider should not ignore signs of eating disorders in Aboriginal adolescents.	1
If the first aider chooses to share their own experience of a mental health problem that is similar to the adolescent's, the first aider should not allow their own story to dominate the conversation.	1
When sharing a personal experience of the same mental health problem (e.g. depression), and there have been difficulties in the first aider's recovery, the first aider should be careful not to convey a bleak attitude towards recovery to the adolescent.	2
If the first aider shares their experience with the adolescent about what worked for them to overcome a mental health problem (e.g. depression), the first aider should emphasise that everyone is different and that the first aider's experience may not apply to them.	2
The first aider should not compare the adolescent's life to their own experiences at that age.	1
If the first aider represents an organisation, they should consider their employer's guidelines on sharing personal information, before disclosing their own personal experience of a mental health problem, with the adolescent.	1

Handling difficulties in the interaction	Round
If the adolescent appears distressed by what they are experiencing, the first aider should reassure the adolescent that help is available.	1
*If the adolescent is being antagonistic or argumentative, the first aider should not respond in a hostile, disciplinary or challenging manner.	1
If the first aider is left feeling bewildered or distressed following a discussion with the adolescent, they should confide their feelings to a trusted friend or health professional, while maintaining the adolescent's privacy.	1
If the adolescent finds it too hard to talk about their problems, the first aider should respect this.	1
*If the adolescent does not want to talk about their problems, the first aider should not take it personally.	1
If the first aider is having trouble engaging or communicating with the adolescent, they should ask whether they would like some help to find someone else to talk to, e.g. a person of a different age or gender.	1
If the first aider is going to recommend the adolescent talk to someone else, then they should explain they are doing this because they think the other person will be of greater help to them and that they are still available to help if needed.	1
If the first aider finds that they have said something in error to the adolescent, they should be upfront and address the error as soon as they can.	1
**Discussing options and getting help**	
The first aider should ensure that the adolescent has had a chance to fully express and explore their issue, before offering possible courses of action, so as not to appear to be minimising or dismissing the problem.	1
The first aider needs to listen attentively and sensitively to the adolescent’s issue in full before they suggest possible courses of action, so as to avoid offering ill-considered or inappropriate advice based on only ‘half the picture’.	1
The first aider should try not to judge a situation on what they would do themselves, but have a discussion with the adolescent about what could be done.	1
The first aider should discuss with and help the adolescent to assess different courses of action and to understand the consequences of each.	1

**Discussing options and getting help (continued)**	
The first aider should ensure that the adolescent has had a chance to fully express and explore their issue, before offering possible courses of action, so as not to appear to be minimising or dismissing the problem.	2
The first aider should recommend that the adolescent talk to the relevant professional (e.g. doctor, counsellor) as early as possible, to talk about what they have been experiencing.	1
The first aider should be aware that the adolescent may not be comfortable using Aboriginal-controlled health services because of concerns about confidentiality and 'shame jobs' if it involves a personal problem.	1
The first aider should be aware of the way shame affects the behaviour of Aboriginal people; for instance, some Aboriginal people may be afraid of attending a mainstream hospital because, historically, being admitted to a hospital with a mental illness caused shame on family and community.	1
The first aider should be aware that the adolescent may not be comfortable using mainstream health services because of fear, language or literacy difficulties, or the racist attitudes of the first contact staff.	1
The first aider should be aware that the adolescent might feel shame in engaging in personal discussions with people of the opposite gender.	1
When encouraging the adolescent to seek professional help, the first aider should ask the adolescent if they would prefer someone of the same gender and culture, if this is possible.	2
The first aider should consider finding out which mental health professionals have been recommended by other people in the adolescent's community.	1
*When assisting the adolescent to find professional help for a mental health problem, the first aider should consider that the adolescent may distrust formal organisations and non-Aboriginal services.	1
If the adolescent resists seeing someone about their problem, offer phone numbers to Lifeline or Kids Help Line or websites, as these are anonymous and may be less confronting.	2
*If the first aider is worried about the adolescent causing harm to self or others, the first aider should seek immediate professional help.	2

Discussing options and getting help (continued)	
The first aider should ask the adolescent who their primary carer is and whether they could be contacted if help is needed.	2
The first aider should find out what informal supports exist in the adolescent's community and encourage use of these where appropriate.	1
In order to encourage the adolescent to participate in positive activities, the first aider should discuss with the adolescent what their interests and activities are.	1
The first aider should be aware that establishing a network of support for the adolescent is a very important step in helping them resolve their mental health crisis, especially if access to professional support or mental health services is limited.	1
If the first aider is worried about the adolescent's safety or the adolescent is experiencing a crisis, then the first aider should be persistent in trying to get the adolescent help and support from others.	1
The first aider should have the adolescent's permission before they seek help from other members of the community, unless they are worried about the adolescent's risk of harm to self or others.	1
The first aider should have the adolescent's permission before they seek help from other members of the community.	1
The first aider should uphold the adolescent's right to confidentiality.	1
The first aider should uphold the adolescent's right to confidentiality, unless the first aider is worried about the adolescent's risk of harm to self or others.	1
*The first aider should be aware that simply reading these guidelines will not equip them to be competent in providing assistance to Aboriginal adolescents.	1
The first aider should be aware that these cultural consideration guidelines are not exhaustive.	1

*Indicates items for which there was 100% endorsement.
