# Supplementary material for: Providing culturally appropriate mental health first aid to an Aboriginal or Torres Strait Islander adolescent: development of expert consensus guidelines
Source: Int J Ment Health Syst. 2014 Jan 28;8:6. doi: 10.1186/1752-4458-8-6 (PMC3922159; doi:10.1186/1752-4458-8-6)
Supplement: Additional file 3 — Items excluded from the guidelines. [file 1752-4458-8-6-S3.pdf]

Items excluded from the guidelines

Understanding cultural influences	Round
*Rather than make assumptions, the first aider should ask the adolescent what influence their culture or background has on their experience of mental health problems.	2
If the first aider plans to approach an adolescent from a different culture about a mental health problem, the first aider should do some research about that culture prior to approaching the adolescent.	1
If the first aider does not have cultural training, then they should not provide mental health first aid to the Aboriginal adolescent.	1
The first aider should not be afraid to clarify with the adolescent what they do not know about the adolescent or their culture.	3
The first aider should avoid referring to deceased relatives of the adolescent.	1
The first aider should be aware that if the adolescent appears angry, irritable or frustrated, it might be a sign of an underlying mental health problem which needs to be addressed.	1
If the family of the adolescent are present, the first aider should expect that they might answer some questions on behalf of the adolescent.	1
*The first aider should ask the adolescent's permission before speaking to family members.	2
*The first aider should be aware that hierarchies of decision-making power within Aboriginal communities may mean that the adolescent is not able to make decisions on their own without first consulting other people.	2
*The first aider should understand how a community might feel shame, for example being asked or forced to do things not in the normal community way, or by keeping contact with someone who is behaving in an unusual manner because of mental illness.	2

**Making the approach**	
If the first aider is Aboriginal they should ask the adolescent about their land/country, cultural background or origin.	2
If the first aider is non-Aboriginal they should introduce themselves in relation to their land/country, cultural background or origin.	1
If the first aider is non-Aboriginal they should ask the adolescent about their land/country, cultural background or origin.	1
The first aider should consult with the relevant respected Elders from the adolescent's community about the right way to help the adolescent with their problem.	1
The first aider should consult with the local respected Elders about the right way to help the adolescent with their problem.	1
*The first aider should not avoid speaking with or offering help to the adolescent out of fear that the first aider may say or do the wrong thing.	2
*The first aider should be aware that it may be easier for the adolescent to talk during an activity that doesn't require a lot of eye contact.	2
The first aider should be aware that the adolescent may find it easier to talk during a time-limited activity that has a definite end point, e.g. while doing the washing up or driving to school.	1
The first aider should use a mutual activity as an opportunity to talk with the adolescent, e.g. going shopping, going for a drive or going for a walk.	1
The first aider should provide an opportunity for the adolescent to choose a mutual activity or not, before discussing personal issues, e.g. going shopping, going for a drive or going for a walk.	1
The first aider should adopt a less formal style of dress to help them engage with adolescents.	1
The first aider should not alter their dress style, as adolescents are happy to seek help from adults who dress differently from them, e.g. wear suits or uniforms.	1
The first aider should dress appropriately according to the adolescent's community because dress codes may vary between different groups.	2
The first aider should be aware that the adolescent may feel more comfortable talking to people of a similar age about their problems or experiences.	1
The first aider should be aware that the adolescent may need to talk to an Aboriginal person because they may relate better and open up more about their problems.	1

Making the approach (continued)	Round
If there is any cultural or gender difference between the first aider and the adolescent, the first aider should comment on this difference and ask the adolescent how they feel about it.	1
If the first aider is a non-Aboriginal person, they should try to get the help of an Aboriginal friend, family member, Aboriginal Elder, or Aboriginal health worker.	1
If the first aider is a non-Aboriginal person, they must enlist the help of an Aboriginal friend, family member, Aboriginal Elder, or Aboriginal health worker.	1
If the first aider is a non-Aboriginal person, they should, with the permission of the adolescent, try to get the help of an Aboriginal friend, family member, Aboriginal Elder, or Aboriginal health worker.	2
If the first aider is a non-Aboriginal person, they should not feel obligated to get the help of an Aboriginal friend, family member, Aboriginal Elder, or Aboriginal health worker, especially in a crisis situation.	2
If the adolescent does not want their Elders or extended family to know what is happening, the first aider should abide by the adolescent's decision.	2
The first aider should be aware that the adolescent may feel more comfortable talking in the presence of friends or family members.	1
The first aider should be aware that the adolescent may feel more at ease about opening up to others who have also experienced mental health problems.	1
If the adolescent feels more comfortable talking to people with similar experiences or people their own age, the first aider should recommend that the adolescent join a support group.	1
If the adolescent asks the first aider for help and the first aider does not know much about the problem, the first aider should learn more about that type of problem.	1
The first aider should express interest in and curiosity about the adolescent.	1

Making the approach (continued)	Round
The first aider should familiarise themselves with technologies that adolescents use, as this may be a way of engaging adolescents more personally and in a way they understand, e.g. with social networking tools such as Facebook, Twitter and YouTube.	1
The first aider should commence speaking to the adolescent with relevant non-threatening statements about the community, e.g. “I saw some footballers here today. I hear they're good. Do you play?”	2
The first aider should engage the adolescent by making general statements such as "I am/people have said they are worried about you" or "are things okay at the moment?".	2
The first aider should focus on engaging with the adolescent and let the adolescent bring up their concerns themselves.	2
If the first aider has been the one to make the approach, they should be straightforward with the adolescent and explain their concerns.	1
Once the first aider has the adolescent's trust, the first aider should tell them they want to help.	2
The first aider should tell the adolescent that they want to support them.	2
Instead of directly offering help, the first aider should show interest in the adolescent.	2
The first aider should engage the adolescent by making general statements such as "things don't look right" or "you don't seem too good in yourself".	1
The first aider should tell the adolescent that they want to help.	1
The first aider should tell the adolescent that they care.	1
If the adolescent looks uncomfortable talking about a particular issue, the first aider should find out why prior to proceeding.	2

**Tips for good communication**	**Round**
*The first aider should express positive attitudes about the adolescent’s culture.	2
*The first aider should be aware that listening, talking and sharing information may take longer with an Aboriginal adolescent than with a non-Aboriginal adolescent.	2
In order to build trust and rapport, the first aider should provide practical support to the adolescent.	1
*The first aider should allow as much time as the adolescent needs to talk.	2
If the first aider has commenced talking with the adolescent about their issues but finds that they are running out of time, the first aider should make another time to talk with the adolescent.	2
If the first aider has established rapport with the adolescent but finds they are running out of time, the first aider should do what they can to stay and continue talking with the adolescent because leaving may result in the adolescent feeling frightened or with the perception that the first aider is untrustworthy, unreliable or uncaring.	2
If the first aider feels that the adolescent is becoming too dependant on them, the first aider should create some time boundaries for the adolescent.	2
The first aider should treat the adolescent as responsible and capable of making decisions.	2
The first aider should adapt their communication style to be more like the adolescent's, e.g. body language, eye contact and the names or words that they use.	1
The first aider should talk to the adolescent as they would to a friend.	1
The first aider should not talk to the adolescent as a friend, but rather as a responsible adult talking to a younger person.	1
*The first aider should avoid trivialising the adolescent's feelings by using statements such as, “When you’re older...” or “Back in my day...” as this may appear dismissive of the adolescent’s perspective or their own experience.	2
The first aider should note any topic the adolescent is finding distressing and avoid asking more about it.	1

Tips for good communication (continued)	Round
The first aider should be aware that it is more important to make the adolescent feel comfortable, respected and cared for, than to do all the 'right things' and follow all the 'rules'.	1
The first aider should not overuse the adolescent’s first name, as this may appear patronising or over-familiar.	1
*The first aider should not use slang or lingo which the first aider is unfamiliar with, as the adolescent will recognise that the first aider is not being genuine.	2
The first aider should use 'I messages' to communicate their thoughts and feelings to the adolescent. 'I messages' are statements that tell the adolescent how the first aider feels about their behaviour and gives the adolescent an understanding of the effects of their behaviour without blaming the adolescent, e.g. "I feel very worried when you... "	1
The first aider should use humour where appropriate.	1
The first aider should avoid asking lots of questions.	1
The first aider should start the conversation by asking closed ('yes' or 'no') questions, so as not to put too much pressure on the adolescent to start opening up.	1
The first aider should use open-ended questions in order to get the adolescent’s input into a conversation. Open-ended questions are questions that can't be answered with a simple 'yes' or 'no', e.g. "How are you feeling about what happened?" instead of "Are you feeling okay about what happened?"	1
*The first aider should not be afraid to ask open, honest questions during the course of discussion.	2
The first aider should not ask the adolescent to justify or explain their behaviour, as asking 'why' can put the adolescent on the defensive.	1
To help the adolescent relax, the first aider should initially do most of the talking.	1
The first aider should encourage the adolescent to do most of the talking.	1
The first aider should not lead the direction of the conversation with the adolescent.	1

Tips for good communication (continued)	Round
If a parent or guardian is present during a discussion with the adolescent and the first aider needs to ask the parent or guardian a question, they should then turn to the adolescent and ask the same question.	1
If a parent or guardian is present during a discussion with the adolescent and the first aider finds someone is reluctant to talk, the first aider should ask to speak with them separately.	1
*If a parent or guardian is present during a discussion with the adolescent, the first aider should ensure that both the adolescent and the parent/guardian have a chance to talk and ask questions.	2
The first aider should allow a larger personal space than is usual for a non-Aboriginal person.	1
The first aider should allow a larger personal space when talking to adolescents of the opposite sex.	1
If the adolescent is distressed, the first aider should convey a sense of comfort by offering a brief hug or touch of the hand.	1
The first aider should be respectful of personal boundaries between themselves and the adolescent, but not to the point of being afraid to show comfort, e.g. by offering a brief hug or touch of the hand.	1
If the first aider wants to give the adolescent a brief hug or touch of the hand etc., they should ask the adolescent if it is alright to do this, especially when they are of a different gender (rather than assume that they want to be touched).	1
* The first aider should be aware that in some instances, it may not be appropriate to give the adolescent a brief hug or touch of the hand, e.g. if they have been sexually abused.	2
The first aider should maintain a comfortable level of eye contact when speaking with the adolescent.	1
Because the adolescent may be uncomfortable with direct eye contact, it is preferable for the first aider to sit or stand side by side with the adolescent looking down at the ground in front of them and making occasional eye contact with the adolescent.	1
The first aider should be aware that the adolescent may not feel comfortable making eye contact with more senior people.	1

Tips for good communication (continued)	Round
The first aider should not seek eye contact from the adolescent unless the adolescent seeks eye contact from them.	2
*The first aider and the adolescent should be positioned at the same level so that they don't have to look up or down at each other.	2
The first aider should sit face to face with the adolescent.	1
The first aider should try to sit facing the same way as the adolescent, looking out rather than directly facing them.	1
When talking with the adolescent, the first aider should not sit behind a desk.	1
The first aider should be aware that a table placed between themselves and the adolescent may be a useful way for the adolescent to avoid feeling exposed to the first aider.	1
The first aider should be aware that a table placed between themselves and the adolescent may be useful for the adolescent to rest their hands on during a conversation.	1
When seated, the first aider should lean forward to show interest when speaking with the adolescent.	
The first aider should sit in a relaxed position when speaking with the adolescent.	1
The first aider should be aware that assuming a relaxed pose when speaking with the adolescent may be interpreted as showing lack of interest in the adolescent's story.	1
If the adolescent is relaxed and open, the first aider should match their body language.	1
The first aider should not make any comments about the adolescent's body language e.g. avoiding eye contact or fidgeting.	1
When speaking with the adolescent, the first aider should allow them to fiddle with personal items if they wish.	**1**
If the adolescent has a lot of physical energy and finds it hard to be still and focus on the conversation, the first aider should provide them with something to do with their hands, e.g. stress ball, paper and pen.	2
*The first aider should notice and acknowledge any non-verbal expression of illness or discomfort by the adolescent.	2

Discussing mental illness with the adolescent	Round
When acknowledging the adolescent's illness or discomfort, the first aider should avoid using mental health terms like 'depressed' or 'anxious' and instead use general statements about ‘‘not being well within themselves’’, or ‘‘not looking too good in themselves”.	1
The first aider should tell the adolescent that they should not feel shame about their mental illness.	1
The first aider should tell the adolescent that having mental health problems is nothing to be ashamed of.	3
The first aider should avoid discussing topics or performing actions that may make the adolescent, their community, or their people feel shame.	1
As a basis for discussion, it is sometimes useful for the first aider to talk about how the adolescent's mental health problem is portrayed in the media, e.g. does the media give a distorted view about their particular problem?	1
The first aider should avoid using ideas about mental illness from television, movies and the media when speaking with the adolescent, as these are often both frightening and inaccurate.	1
The first aider should avoid using ideas about mental illness from television, movies and the media when speaking with the adolescent, as these may not reflect what the adolescent believes or feels.	1
If done with care, it is sometimes useful for the first aider to refer to ideas on television, movies and the media as a basis for discussion to explore the adolescent's fears and help them gain an understanding of their own experience.	1
The first aider should encourage the adolescent to talk to them about their experiences and beliefs about mental illness.	1
The first aider should reassure the adolescent that they may feel better once they have spoken about their problems.	1
The first aider should reassure the adolescent that sharing their story may help them to feel better.	2
*The first aider should avoid telling the adolescent what they should do to solve their problems.	2
The first aider should ask permission to give opinions or offer advice so that it is not seen as criticism or taken defensively.	1
If the first aider is uncertain whether the adolescent wants guidance, they should ask them directly.	1
The first aider should avoid steering the conversation toward the answer they think will 'solve' the adolescent's problems.	1

Discussing mental illness with the adolescent (continued)	Round
If the first aider has personal experiences of mental health problems, they should share these with the adolescent, to normalise the experience for them.	1
If the first aider has personal experiences of mental health problems, they should not share these with the adolescent.	1
If the first aider has personal experiences of mental health problems, they should gauge whether or not it may be helpful to share these with the adolescent on an individual basis.	1
Before the first aider chooses to share their own personal experience of a similar mental health problem with the adolescent, they should be aware that the adolescent may feel as though their own problem is not important because others have been through it before.	1
Before self-disclosing, the first aider should reflect on why they wish to share their experience with the adolescent and proceed only if it is for the sake of the adolescent.	2
If the first aider chooses to share their own experience of a mental health problem that is similar to the adolescent's, they should ask the adolescent whether they want to hear it before going ahead.	1
The first aider should not share their experience of a mental health problem as a way of suggesting to the adolescent what they should do.	1
If the adolescent appears distressed by what they are experiencing, the first aider should normalise it by saying it is common for adolescents to feel this way.	1
**Handling difficulties in the interaction**	
If the adolescent is finding it difficult to express their emotions, the first aider should offer a few terms to pick from to help the adolescent find the language they are looking for. For example, "To me, you don't look very happy. Are you feeling sad, or angry, or frustrated?"	1
If the adolescent is distressed, the first aider should convey a sense of comfort by using a few appropriate words, e.g. "It's okay, I'm here."	1

Handling difficulties in the interaction (continued)	Round
If the adolescent appears agitated, the first aider should offer to take them outside for a walk while talking with them.	1
If the adolescent is expressing anger, the first aider should offer empathy and try to find out why they are feeling angry.	2
If the adolescent is expressing excessive anger or behaving aggressively, the first aider should clearly state that they cannot communicate with the adolescent if they are shouting or acting in a threatening way.	1
If the first aider feels uncomfortable during their discussion with the adolescent, they should admit it.	1
If the first aider feels uncomfortable during their discussion with the adolescent, they should ask the adolescent if they are uncomfortable also.	1
If the first aider feels uncomfortable with what the adolescent is saying, they should suggest that the adolescent find someone else to speak to and ask if they would like any assistance with this.	1
If the first aider feels startled or disturbed by what the adolescent says, the first aider should reflect back to the adolescent how they feel.	1
If the first aider feels startled or disturbed by what the adolescent says, the first aider should reflect back to the adolescent how they feel, and ask the adolescent whether they have ever felt a similar way.	1
If the adolescent makes negative comments about the first aider or what they have said, the first aider should not take these personally.	2
If the adolescent cannot or does not want to talk about what they are experiencing, the first aider should respect this and not pry.	1
*If the adolescent is suspicious or expresses resentment towards the first aider because they are from a different culture, the first aider should empathise with the adolescent and reassure them that they are there to help.	2
If the first aider is having trouble interpreting the adolescent's behaviour, they should ask them directly to explain what they are doing.	1
If the adolescent appears to be non-communicative, difficult to engage or the first aider is unable to connect with them, the first aider should consider assisting the adolescent to find someone more suitable to help them.	1
If the adolescent is infringing on the first aider's personal space, the first aider should talk to them directly about it.	2

Discussing options and getting help	Round
The first aider should ask the adolescent if they wish to speak to an Aboriginal mental health worker.	2
*The first aider should help the adolescent find a mental health worker who will collaborate with an Aboriginal mental health professional to ensure that their diagnoses and treatment plans are culturally appropriate.	2
The first aider should ask the adolescent's family to get involved in supporting the adolescent while they get better.	1
Where possible, the first aider should assist the adolescent experiencing mental health problems to speak to an Aboriginal mental health worker.	1
If the first aider is worried about the adolescent causing harm to self or others, the first aider should seek immediate professional help, including the police.	2
The first aider should encourage activities that strengthen the adolescent's connection to culture.	2
If the first aider is worried about the adolescent's safety, or the adolescent is experiencing a crisis, then the first aider should ask the adolescent's family to get involved in supporting them while they get better.	1
If the adolescent tries to push the first aider away, the first aider should be persistent in trying to get the adolescent help and support from others.	1
If the adolescent wants to be left alone, and can assure the first aider of their safety, the first aider should agree.	1
The first aider should offer practical assistance where appropriate, e.g. help them with legal, housing or custody matters.	2
If the adolescent is afraid that they may suffer from 'payback', the first aider should encourage them to seek appropriate help from a traditional healer, respected Elder or family member.	2
If the first aider knows that there are positive family dynamics within the adolescent's family, the first aider should ask the adolescent's family to get involved in supporting the adolescent while they get better.	2

* Indicates items excluded on the basis of falling into the ‘re-rate’ category after two Rounds.
