# Supplementary material for: Providing culturally appropriate mental health first aid to an Aboriginal or Torres Strait Islander adolescent: development of expert consensus guidelines
Source: Int J Ment Health Syst. 2014 Jan 28;8:6. doi: 10.1186/1752-4458-8-6 (PMC3922159; doi:10.1186/1752-4458-8-6)
Supplement: Additional file 4 — Communicating with an Aboriginal or Torres Strait Islander Adolescent: Guidelines for being culturally appropriate when providing mental health first aid. [file 1752-4458-8-6-S4.pdf]

COMMUNICATING WITH AN ABORIGINAL OR TORRES STRAIT ISLANDER ADOLESCENT:

GUIDELINES FOR BEING CULTURALLY APPROPRIATE WHEN PROVIDING MENTAL HEALTH FIRST AID

Purpose of these guidelines

These guidelines describe how members of the public should tailor their approach when providing mental health first aid to an Aboriginal or Torres Strait Islander adolescent who may be developing a mental illness or experiencing a mental health crisis. The role of the mental health first aider is to assist the young person until appropriate professional help is received or the crisis resolves. These guidelines include information on how first aiders can be respectful of cultural differences when assisting young people.

This document does not cover how to provide mental health first aid for specific mental illnesses or mental health crises. Additional useful information can be found in:

- *Youth Mental Health First Aid: A Manual for Adults Assisting Young People*
- *Aboriginal and Torres Strait Islander Mental Health First Aid Manual*
- *Cultural Considerations & Communication Techniques: Guidelines for Providing Mental Health First Aid to an Aboriginal or Torres Strait Islander Person*
- *Depression: Guidelines for Providing Mental Health First Aid to an Aboriginal or Torres Strait Islander Person*
- *Psychosis: Guidelines for Providing Mental Health First Aid to an Aboriginal or Torres Strait Islander Person*
- *Suicidal Thoughts & Behaviours and Deliberate Self-Injury: Guidelines for Providing Mental Health First Aid to an Aboriginal or Torres Strait Islander Person*
- *Trauma and Loss: Guidelines for Providing Mental Health First Aid to an Aboriginal or Torres Strait Islander Person*
- *Problem Drinking: Guidelines for Providing Mental Health First Aid to an Aboriginal or Torres Strait Islander Person*
- *Problem Drug Use: Guidelines for Providing Mental Health First Aid to an Aboriginal or Torres Strait Islander Person*

Specific training on how to provide mental health first aid to young people is available in the 14-hour Youth Mental Health First Aid course. Specific training on how to provide mental health first aid to Aboriginal or Torres Strait Islander people is available in the 14-hour Aboriginal and Torres Strait Islander Mental Health First Aid course.

You can purchase manuals, download guidelines and read about these courses at the Mental Health First Aid Australia website, www.mhfa.com.au

Development of these guidelines

The following guidelines are based on the expert opinions of Aboriginal mental health professionals from across Australia, who have extensive knowledge of, and experience in, the mental health of Aboriginal youth.

Although these guidelines are copyright, they can be freely reproduced for non-profit purposes provided the source is acknowledged. Enquiries should be sent to Mental Health First Aid Australia: mhfa@mhfa.com.au

This research was funded by the Australian Government Department of Health and Ageing.

How to use these guidelines

It is important to acknowledge that Aboriginal and Torres Strait Islander communities are not all the same; they may differ in their understanding, interpretations, approaches and treatment of mental illness. Be aware that the community that the adolescent lives in may not view mental illness in the same way that you do.

In these guidelines the word *Aboriginal* is used to represent all Australian Aboriginal and Torres Strait Islander people.

In these guidelines the word *adolescent* refers to an Aboriginal or Torres Strait Islander adolescent. There are a number of ways of defining adolescence and this may differ between communities. Here adolescence is defined as those aged between 12 and 18. However, adolescence can start earlier than 12 years and can continue through to the early 20s, so items in these guidelines could be relevant when helping people who are a little younger or older. When providing mental health first aid to an adolescent, first aiders need to use good judgment about whether the information is going to be appropriate for helping a person outside of the age range specified.

These guidelines are a general set of recommendations about how you can best communicate with an Aboriginal adolescent who may be experiencing a mental illness or developing a mental health crisis. Each individual is unique and it is important to tailor your support to the adolescent's needs. These recommendations therefore may not be appropriate for every adolescent. Be aware that these guidelines are not exhaustive and simply reading them will not equip you to be competent in providing assistance to Aboriginal adolescents.

Understanding cultural influences

Be aware of the impacts of culture and history

Social, cultural and historical factors all have an impact on the health and wellbeing of Aboriginal and Torres Strait Islander people. You should be aware of the

adolescent's cultural background, local cultural norms and the hierarchy of decision-making power within their community. It is important to recognise that there are cultural differences among Aboriginal and Torres Strait Islander communities.

You should have a basic awareness of the historical invasion of Australia by Europeans and an understanding of the ongoing impact on the health and wellbeing of Aboriginal and Torres Strait Islander people.

If you are providing mental health first aid outside your own culture or community you should be culturally competent and practice cultural safety (see box below). You should not express any negative opinions about the adolescent's culture.

It is important to recognise that no one is ever entirely culturally competent, as culture changes and evolves with time. Even if you have not undertaken cultural awareness training (see box below), you should still provide mental health first aid to the Aboriginal adolescent, because any support, especially in a crisis, is better than none.

CULTURAL AWARENESS

Cultural awareness involves recognising that we are all shaped by our cultural background, which in turn influences how we interpret the world around us, perceive ourselves and relate to other people. It includes acknowledging past histories, policies and practices.

CULTURAL COMPETENCE

Cultural competence focuses on the capacity of a person to apply cultural awareness and knowledge to their behaviours and attitudes.

Being culturally competent involves behaviours and attitudes that reflect an awareness about:

- How a person's culture shapes their behaviour and how they understand health and ill-health
- The specific cultural beliefs that surround mental illness in a person's community
- How mental illness is described in a person's community, e.g. knowing what words and ideas are used to talk about the symptoms or behaviours
- Which concepts, behaviours or language are taboo and may cause shame (see the definition of 'shame' below).

SHAME
The feeling of shame for an Aboriginal or Torres Strait Islander person is not easily defined and bears little or no resemblance to a dictionary definition. Shame can occur when a person is singled out or in a circumstance that directly targets a person's dignity. Shame may be felt as a result of:

- a lack of respect
- embarrassment
- self importance/self promotion
- rudeness
- a breach of accepted Aboriginal "norms" and/or taboos

A **shame job** is an an event which causes a person shame or embarrassment.

The concept of shame is very important within many Aboriginal and Torres Strait Islander communities. Shame can be overwhelming, disempowering and can also act as a barrier to seeking help.

CULTURAL SAFETY
Practicing cultural safety involves:

- Respecting the culture of the community by using appropriate language and behaviour
- Never doing anything that causes the person to feel shame
- Supporting the person's right to make decisions about seeking culturally-based care.

Learn about the adolescent's cultural beliefs and concept of mental illness

A person's culture plays a very important role in the way they understand and talk about mental health and mental illness, and how they go about seeking help from friends, family or professionals. There are also differences in the way that communities and individuals think about mental health. In providing mental health first aid, you should be aware that your concept of mental health may differ from the adolescent's and, therefore, you should learn about the specific cultural beliefs that surround mental illness in the adolescent's community. This includes being aware of the concept of mental illness within the adolescent's community, including symptoms and behaviour, and the terminology used. You should consider that the adolescent might understand mental health within a wider context of health and wellbeing, which includes how the adolescent functions socially and emotionally in their community. The adolescent may see behavioural signs of mental illness as part of a person's spirit or personality and may not think of these as symptoms of a mental illness.

When assisting the adolescent you should be aware that certain cultural experiences of Aboriginal people (such as seeing spirits or hearing voices of recently deceased loved ones) may be misdiagnosed or mislabelled as symptoms of mental illness. Previous misdiagnosis of a mental health problem within the Aboriginal adolescent's community could be a barrier to help seeking. For these reasons, you should take into consideration the spiritual and cultural context of the adolescent's behaviours.

On the other hand, you should not assume that unusual or out-of-character behaviours are a part of the adolescent's culture, as they may be signs of mental health problems. Before acting on any assumptions it is important to explore these signs.

Be aware of challenges the adolescent might be experiencing

When providing mental health first aid to the adolescent you should be aware that they may have additional challenges because of social problems such as racism and discrimination. The adolescent may carry a lot of anger from past injustices that they or their family have endured and you should consider this in your approach.

If the adolescent appears angry, irritable or frustrated, you should not automatically assume it is a sign of an underlying mental health problem. The adolescent might be expressing these feelings due a range of other factors, e.g. transgenerational trauma, social disadvantage, racism and discrimination.

Be aware that the adolescent is likely to have experienced the death of a family member, community member or friend. In fact, the adolescent may have experienced the death of more than one loved one in a short space of time, making the recovery process from each loss very difficult. It is important to be aware of how the adolescent's family deals with death and grieving because this will vary between regions and families, e.g. some communities believe that mentioning the names of the deceased or displaying their photograph will call the person's spirit back and not let them pass on.

Address the death of a person in a sensitive manner and be prepared to be led by the adolescent when discussing loss and any deceased relatives or friends. Also, when discussing death and a loss experienced by the adolescent, be aware that suppressed emotions may come to the surface.

Think about the impact that family may have on the adolescent

Recognise the cultural significance of family and the importance to the adolescent of strong family ties. Do not criticise members of the adolescent's extended family. Be aware that, because of the significance of family within Aboriginal communities, problems within the family can have a greater impact on the adolescent.

Understand what might cause the adolescent to feel shame

Be aware of the cultural concept of shame within the adolescent's community. You should understand what might cause the adolescent to feel shame, such as topics or behaviours that may be considered 'mad', abnormal, unusual or embarrassing, and do your best to avoid these. You should also know how an Aboriginal adolescent might feel community shame. For example, do not talk about the adolescent's mental health problems in front of other community members. In some communities the stigma around mental health is strong, so you need to be sensitive and careful when

approaching an adolescent and their family who may be uninformed about mental health issues.

Although it is important to be aware of the historical factors that may lead to shame, you should approach the adolescent with an open mind and be careful not to push this previous trauma upon them.

Making the approach

Approach the adolescent in a sensitive and appropriate manner

An introduction may be needed between yourself and the adolescent. If this is the case, you should be aware that when Aboriginal people introduce themselves, they may do this in relation to their land/country, cultural background or origin. If you are Aboriginal yourself, you should introduce yourself in this way. Whether or not you are Aboriginal, you should offer your first name to create a less formal atmosphere. Using titles such as 'doctor' or 'mister' creates a hierarchy, and the adolescent may perceive you as wanting power over them. Do not be overly assertive or 'big-note' yourself when talking with the adolescent because it is not considered polite in many Aboriginal communities.

You should make sure that you approach the adolescent privately about their experiences, at a time and place that is convenient and free of distractions, and when you have plenty of time for the discussion. Consider that the adolescent may feel more able to discuss their problems when no one else is listening. Find out where the adolescent feels comfortable or safe to talk, as they are more likely to engage with you in a setting that is within their comfort zone, e.g. in a café or at home.

You could ask the adolescent if they wish to do a mutual activity whilst talking. If you do this, you should ensure that you inform the adolescent that it is for the purpose of having a chat (so they don't feel pressured or hemmed in), e.g. "Would you like to go for a walk with me so that we can talk?"

Remember that building a trusting relationship where the adolescent feels comfortable is more important than other factors (e.g. how you introduce yourself or the place of discussion).

Know how to handle concerns about cultural or gender differences

Although some adolescents prefer confiding in people of their own cultural background, don't assume that this is always the case; ask if they would prefer this. Be aware that gender and cultural differences between yourself and the adolescent might be exacerbated by discussing private issues, such as commenting on the quality of family relationships, discussing intimate relationships, and, most particularly, topics that include any issues of a sexual nature. If the adolescent shows any concern about a cultural or gender difference between the two of you, you should explore the possibility of getting help from someone the adolescent feels more comfortable with.

Ask the adolescent who they wish to involve in discussions

Because family and friends are a very big part of Aboriginal and Torres Strait Islander cultures, you should anticipate that family or friends may expect to be involved in caring for the adolescent. If family or friends express that they wish to be involved,

you should make sure the adolescent is okay with this. Allow the adolescent to choose who they talk to and who is present in these discussions.

If the family of the adolescent are present, avoid asking the adolescent questions that might cause the adolescent embarrassment. You should also ensure the adolescent has the opportunity to answer any questions, even though the family may answer for them.

If you can't help, ensure someone else does

You may find yourself in the situation where the adolescent asks you for help and you do not know much about the problem. In this instance, you should still try to support the adolescent and assist them to get other help. If you do suggest that the adolescent speak to someone else about their problem, you should find a suitable replacement, rather than leaving this task for the adolescent, e.g. "I don't know if I can offer you the best advice on X, but I can help you find someone else to talk to."

Similarly, if the adolescent doesn't feel comfortable talking to you, you should help them to find a more suitable person to talk to.

Engage the adolescent before discussing personal issues

Before discussing personal issues with the adolescent, you should take the time to engage with them first (e.g. getting them to talk about their interests and social life). If you don't know what the adolescent is interested in, you should try to learn more from them. Be aware that your reactions to the adolescent's 'everyday problems' may influence what else the adolescent decides to share.

Keep in mind that some adolescents (especially boys) may fear opening up about their problems in case their vulnerability is labelled as a weakness. You should avoid pressuring the adolescent to talk. Tell them they don't have to talk until they are ready to do so, but that you will listen to them when they are. Also let them know that the conversation will remain private unless they talk about harming themselves or someone else.

Be aware that sometimes adolescents struggle to ask for assistance, or reject help when offered, even if they feel that a situation is out of control. The adolescent may hide or play down their problem if they are worried about upsetting or disappointing you. It is also possible that the adolescent does not like being the focus of attention. Do not presume that the adolescent doesn't want your help, even if their initial reaction is negative. The adolescent may need more than one conversation to open up about what is bothering them. You should not give up on trying to engage with the adolescent if they are finding it difficult to open up, but rather try again another time.

Because the adolescent may not wish to open up until they feel you care enough, are trustworthy and are willing to listen, make sure that you take the time to build rapport and trust with them. Be prepared for this process to take longer if you are not Aboriginal or if the adolescent has disengaged from other people. Once you have the adolescent's trust, you should tell them that you want to support them.

Tips for good communication

Make the adolescent the focus of the interaction

When communicating with the adolescent, give them your full attention. Set aside your own issues and try to focus on the adolescent's concerns. Ask the adolescent to explain their experiences and how they feel about them rather than making your own interpretation. Be sure to talk 'with', not 'at', the adolescent and avoid sounding condescending or patronising.

Be warm and non-judgmental

Be warm, caring and non-judgmental toward the adolescent. Offer the adolescent consistent emotional support and understanding and help them feel more positive about themselves. Avoid stereotyping them, e.g. "Teenagers are always so difficult." You should treat the adolescent with respect and fairness, and avoid confronting, criticising or blaming them. Do not contradict or minimise the adolescent's feelings by using statements such as "You're not depressed, you're just bored." Instead, acknowledge the adolescent's expertise about their own life and try to empathise with how they feel.

Offer positive feedback to the adolescent, as this may encourage them to communicate with you, e.g. "I think it's great that you are willing to talk to me about this." Convey a message of hope to the adolescent by assuring them that help is available and things can get better.

Be honest, reliable and consistent

Be honest during the interaction. It is important to be reliable and consistent in your behaviour with the adolescent. Adolescents are very good at reading an adult's attitude and are particularly tuned in to anyone who is faking it. Therefore, it is important that you are genuine by being yourself. Do not make any promises to the adolescent that you can't keep. If you find that you have said something in error to the adolescent, you should be upfront and address the error as soon as you can.

Adapt your communication style

It is important that you are aware of respectful ways to communicate with the adolescent, including body language, seating position and use of certain words. This may differ between communities and regions. You should recognise that each adolescent's situation and needs are unique. Rather than automatically adopting communication styles based on assumptions, you should pay attention to what the adolescent feels comfortable with and use this to guide your communication.

Talk to the adolescent in a calm and confident manner

You should show a confident manner when interacting with the adolescent. Use a calm voice and steady tone. Never raise your voice if you can help it.

Use clear and simple language

When talking with the adolescent, you should use simple and clear language. If you realise you are using language that the adolescent does not understand (e.g. metaphor or humour), you should change your approach and use direct language.

Be aware that some adolescents do not communicate well verbally, and it is important to adapt to the adolescent's preferred communication style, e.g. using art if they do not feel comfortable with spoken language. If the adolescent has difficulties with communication (e.g. vision impairment, cognitive impairment, poor literacy), you should do your best to adapt your communication to meet these needs. Try to be aware of when the adolescent is not listening to you and respond by changing the way you say or do things.

Let the adolescent tell their story

As far as possible, you should let the adolescent set the pace and style of the interaction. Listen to the adolescent without interrupting them. Allow the adolescent the opportunity to 'have a yarn' or 'tell the story' as this may work better than asking 'yes' or 'no' questions. The adolescent may not respond to a question with a direct answer. Asking for a clear response to a question of a personal nature may result in the adolescent feeling shame.

You should make sure you actively listen and ask relevant questions to check your understanding and acknowledge that you have heard what the adolescent has said. Additionally, make a conscious effort to listen for the feelings and meaning behind the adolescent's words, and respond to this. For example, if an adolescent says, "There's no point in going to school anymore", it could mean that they feel that the future is hopeless.

Allow periods of silence while the adolescent considers their response to a question. After speaking, you should be patient and allow plenty of time for the adolescent to collect their thoughts, reflect on their feelings, and decide what to say next. If you often use long silent pauses yourself, you should explain that you are just thinking about the adolescent's options, as the adolescent may otherwise misinterpret this negatively.

If a parent or guardian is present during the conversation, you should encourage turn-taking and courteous silence when another person is speaking. If you are having a private discussion with the adolescent and other people arrive, you should take a moment to ask the adolescent in private what they would like to do, e.g. continue the discussion in front of others, ask others to leave or make another time to continue your discussion.

Be aware of body language

Be conscious of your body language and what this conveys when communicating with the adolescent, e.g. posture, facial expressions and gestures. You should use cues like nodding to keep a conversation going with the adolescent and avoid negative body language such as crossing your arms, putting your hands on your hips or looking uninterested. Also avoid distracting gestures, such as fidgeting with a pen, glancing at other things or tapping your feet or fingers, as these could be interpreted as lack of interest. Consider that the adolescent may be uncomfortable with direct eye contact.

Be conscious of the adolescent's body language as well, as this can provide clues to how they are feeling or how comfortable they feel talking with you. If the adolescent appears defensive, you should make your body language as open as possible, e.g. by appearing relaxed, sitting alongside the adolescent but angled toward them and keeping your voice calm and low.

Provide a comfortable and appropriate amount of personal space

When talking with the adolescent, you should notice how much personal space they feel comfortable with and not intrude beyond that. Consider the possible consequences of physical contact with the adolescent, as it may cause problems with personal boundaries and may lead to legal troubles. If you represent an organisation (e.g. if you are a teacher), you should consider you employer's guidelines on physical contact with an adolescent before giving the adolescent a brief hug or touch of the hand.

Discussing mental illness with the adolescent

Let the adolescent tell you about their experiences and beliefs

Talk openly with the adolescent about mental illness, adapting your language to their age and maturity. You should be aware that the adolescent may not use mental health terms when communicating that they are feeling mentally unwell, e.g. "I feel like crap" rather than "I feel depressed" or "I'm feeling anxious". When acknowledging the adolescent's illness or discomfort, use the words that they use to describe their problems.

Be aware that the adolescent may hold stigmatising attitudes towards mental illness and be careful not to communicate such attitudes yourself. Don't use labels the adolescent may find stigmatising, e.g. 'mentally ill', 'drug addict'.

Allow the adolescent to talk to you about their experiences and beliefs about mental illness if they want to. Ask the adolescent's permission before asking questions about sensitive topics, e.g. "I would like to talk about something important, but I'm aware it might be painful for you. Is that okay?" Let the adolescent speak and tell their story first, and then help them after they have said their piece. Tell the adolescent that they have done the right thing in talking to someone about their problems.

Do not use scare tactics or threats when talking to the adolescent, e.g. "If you keep thinking like this, you'll end up in big trouble." Never tell the adolescent to "snap out of it" or "stop thinking that way".

Eating disorders in Aboriginal adolescents

Although some people believe that Aboriginal adolescents are not affected by eating disorders and body dissatisfaction, you should be aware that this is a myth. Therefore, you should not ignore signs of eating disorders in Aboriginal adolescents. Additional useful information can be found in the Mental Health First Aid Australia *Eating Disorders: First Aid Guidelines.*

Know how to share your own experiences of mental illness

If you choose to share your own experience of a mental health problem that is similar to the adolescent's, do not allow this to dominate the conversation. Do not compare the adolescent's life to your own experiences at that age. If you talk about what worked for you to overcome a mental health problem (e.g. depression), you should

emphasise that everyone is different and that your experience may not apply to them. On the other hand, if there have been difficulties in your recovery, you should be careful not to convey a bleak attitude towards recovery to the adolescent.

If you represent an organisation, you should also consider your employer's guidelines before disclosing your own personal experience of a mental health problem to the adolescent.

Discussing options and getting help

Offer the adolescent options and assistance in finding a solution to their problems

You should not imply to the adolescent that simply talking to you about their mental health problems will make these problems go away. Try to help the adolescent find solutions without trying to fix their problems for them. Offer options for actions that could help with their problems and allow for compromise to give the adolescent a sense of control. You should discuss with and help the adolescent to assess different courses of action and to understand the consequences of each.

Listen attentively and sensitively to the adolescent's problems in full before you suggest possible courses of action. Otherwise you could be offering ill-considered or inappropriate advice based on only 'half the picture', or you might appear to be minimising or dismissing their problems. You should try not to offer a solution based on what you would do yourself, but have a discussion with the adolescent about what could be done.

If you are concerned about the adolescent's safety (e.g. they are experiencing abuse or bullying), you should ask them directly about this and reassure them that you want to keep them safe. If you are worried about the adolescent causing harm to self or others, you should seek immediate professional help.

Recommend that the adolescent talk to a professional as soon as possible

If the adolescent appears distressed by what they are experiencing, you should reassure the adolescent that help is available. You should recommend that the adolescent talk about what they have been experiencing to a relevant professional (e.g. doctor, counsellor) as early as possible. When you encourage the adolescent to seek professional help, you should ask them what they prefer. You might also consider finding out which mental health professionals have been recommended by other people in the adolescent's community.

Be aware that the adolescent may distrust formal organisations or non-Aboriginal services. For example, the adolescent might prefer not to use mainstream health services because of the way shame affects the behaviour of Aboriginal people. Some may be afraid of attending a mainstream hospital because, historically, being admitted to a hospital with a mental illness caused shame on family and community. Another possibility is that the adolescent feels uncomfortable using these services because of fear, language or literacy difficulties, or the racist attitudes of the first contact staff.

On the other hand, you should also be aware that some adolescents are not comfortable using Aboriginal-controlled health services because of concerns about confidentiality and shame jobs.

You should also be aware that the adolescent might feel shame in engaging in personal discussions with people of the opposite gender. Ask the adolescent if they would prefer someone of the same gender and culture, if this is possible.

If the adolescent resists seeing someone about their problem, you should offer the adolescent phone numbers to Lifeline or Kids Help Line, or website addresses, as these are anonymous and may be less confronting.

Encourage the use of other supports in the adolescent's community

You should find out what informal supports exist in the adolescent's community and encourage use of these where appropriate. Try to find out who has the most positive influence on the adolescent and who the adolescent will respect and listen to. Ask the adolescent who their primary carer is and whether they could be contacted if help is needed.

You should have the adolescent's permission before you seek help from other members of the community, unless you are worried about the adolescent's risk of harm to self or others. Similarly, you should uphold the adolescent's right to confidentiality, unless you are worried about the adolescent's risk of harm to self or others.

Encourage the adolescent to participate in positive activities in their community. In order to help them find suitable activities, you should ask about their interests.

In a crisis

If you are worried about the adolescent's safety or if the adolescent is experiencing a crisis, then you should be persistent in trying to get the adolescent help and support from others. Be aware that establishing a network of support for the adolescent is a very important step in helping them resolve their mental health crisis, especially if access to professional support or mental health services is limited.

If you are concerned the adolescent is suicidal or harming themselves, you should consult the following guidelines: *Suicidal Thoughts & Behaviours and Deliberate Self-Injury: Guidelines for Providing Mental Health First Aid to an Aboriginal or Torres Strait Islander Person.*

If you are concerned the adolescent has had a traumatic experience, you should consult the following guidelines: *Trauma and Loss: Guidelines for Providing Mental Health First Aid to an Aboriginal or Torres Strait Islander Person.*

Handling difficulties in the interaction

You may have problems engaging and communicating with the adolescent or they may not want to talk about their problems. If this is the case, you should respect this and not take it personally. You may have to consider finding someone else to help the adolescent. Ask whether they would like some help to find someone else to talk to,

e.g. a person of a different age or gender. If you are going to recommend the adolescent talk to someone else, then you should explain that you are doing this because you think the other person will be of greater help to them and that you are still available to help if needed.

You should note any topic the adolescent finds distressing and give them time to think and the opportunity to continue the conversation after a pause. If the adolescent is being antagonistic or argumentative, you should not respond in a hostile, disciplinary or challenging manner. If you feel startled or disturbed by what the adolescent says, you should remain neutral, e.g. in your phrasing, vocal tone and body language.

Exercise self-care

Following a discussion with the adolescent you may be left feeling bewildered or distressed. If this occurs you should confide your feelings to a trusted friend or health professional, while maintaining the adolescent's privacy.

Although these guidelines are copyright, they can be freely reproduced for non-profit purposes provided the source is acknowledged.

Please cite these guidelines as follows:

Mental Health First Aid Training and Research Program. (2013). *Communicating with an Aboriginal or Torres Strait Islander adolescent: Guidelines for being culturally appropriate when providing mental health field.* Melbourne: Mental Health First Aid Australia.
